# Supplementary material for: Step-by-Step Replacement of Cyano Groups by Tricyanovinyls—The Influence on the Acidity
Source: Molecules. 2023 Dec 18;28(24):8157. doi: 10.3390/molecules28248157 (PMC10745934; doi:10.3390/molecules28248157)
Supplement: Supplementary file 1 [file molecules-28-08157-s001.zip › updated-molecules-2711695-supplementary.pdf]

# SUPPLEMENTARY MATERIAL

*of the Article*

# Step-by-Step Replacement of Cyano Groups by Tricyanovinyls — The Influence on the Acidity

**Agnes Kütt**

Institute of Chemistry, University of Tartu, Ravila 14A, Tartu, 50411, Estonia; agnes.kutt@ut.ee

## 1. Materials and Methods

### *1.1. Computational Gas-phase acidities*

All electronic structure calculations in this study were performed with the quantum chemical program package Turbomole version 7.2 or Gaussian 16.

For all the compounds, frequency calculations were carried out to check for the presence of imaginary frequencies. These may indicate that found geometry corresponds to a transitional state rather than energy minimum. However, small negative frequencies may be an artifact of computational procedure. In some cases, imaginary frequencies were found, and geometries were adjusted and reoptimized to eliminate or reduce them. It was found that the effect of such corrections on the computed GA values was negligible.

Calculating the molecule as a cycle where the proton is attached between two CN groups did not lead to any stable species. During the geometry optimization, the ring is broken, or a structure with imaginary frequencies is obtained.

In the case of Gaussian B3LYP/6-311+G(d,p) results, the *H* and *G* values come straight from the output as "Sum of electronic and thermal Enthalpies" and "Sum of electronic and thermal Free Energies", respectively. In the case of G4(MP2) method, the H and G value comes from the output as "G4MP2(0 K)" and "G4MP2 Energy", respectively. W1RO and CBS-APNO final energies come from Gaussian outputs as "W1RO   Enthalpy" and "W1RO   Free Energy", and "CBS-APNO Enthalpy" and "CBS-APNO Free Energy", respectively. Gaussian calculations are performed at the temperature 298.2 K and at the pressure 1.0 atm.

In the case of Turbomole results, the *H* and *G* values need to be calculated for every species according to equations 2-3. Thermochemical properties (Enthalpy and Chemical potential) are available from the frequency calculations, where the following parameters are used: temperature 298.15 K, pressure 0.1 MPa, frequency scale 0.9914, sigma 1.0, symmetry C1.

$$H = \text{total energy} + \text{Enthalpy} \quad (S1)$$

$$G = \text{total energy} + \text{Chemical potential} \quad (S2)$$

Then, PA and GA are calculated as follows:

$$\text{PA} = H(\text{A}^-) + H(\text{H}^+) - H(\text{AH}) \quad (S3)$$

$$\text{GA} = G(\text{A}^-) + G(\text{H}^+) - G(\text{AH}) \quad (S4)$$

Where values for proton are: $H(\text{H}^+)$ = 1.481 kcal/mol; $G(\text{H}^+)$ = -6.275 kcal/mol.

*1.2. Computational solution phase acidities*

Acidities in the solution were estimated using either a correlation between p$K_a$ values in two solvents or values computed with the COSMO-RS method and corrected using the available experimental data for similar compounds. The auxiliary acids that were chosen to correct computational p$K_a$ values are all cyanocarbon acids measured in 1,2-dichloroethane (DCE). The obtained results are summarized in Table 4 in the main text and Table S8.

The COSMO-RS files were calculated for all the conformers of anions and all the conformers/isomers of the most stable tautomer of neutral form. For this, two sequenced COSMO calculations were performed. First, the structure was optimized in the ideal conductor using the BP86/def-TZVP basis set, and then the COSMO file with the FINE cavity parameter was calculated using the BP86/def2-TZVPD method. Using obtained cosmo files, a conformer set was created for anion and neutral species, and p$K_a$ values in MeCN, DMSO, and $H_2O$ were calculated using COSMOtherm software with parametrization BP_TZVPD_FINE_19. All unique conformers were considered. The conformational search was conducted only for anion **14** with COSMOconf software using BP-TZVP-GAS methods.

p$K_a$(DMSO) values in the current work are obtained from p$K_a$(MeCN) values by averaging two different values calculated from previously published p$K_a$(MeCN) vs p$K_a$(DMSO) correlation equations.[34,42 in the main text] Also, COSMO-RS p$K_a$(DMSO) values are available in Table S8. To correlate the assigned p$K_a$(DMSO) values with pKa(DMSO) COSMO-RS values, a very good correlation is obtained, however, the values themselves deviate from each other. The equation is as follows:

$$\text{p}K_a\text{(DMSO) ASSIGNED} = 1.299(0.015) \cdot \text{p}K_a\text{(DMSO) COSMO-RS} - 0.63(0.20) \quad \text{(S5)}$$

$$r^2=0.998;\ S=0.685;\ n=19$$

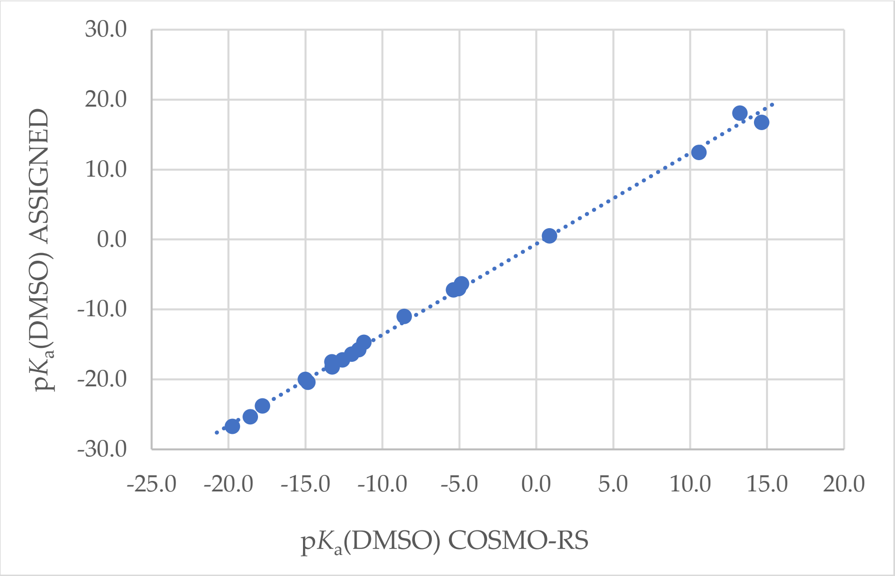


**Figure S1.** Correlation between p$K_a$(DMSO) values listed in Table S4 and computationally obtained COSMO-RS p$K_a$(DMSO) values according to Eq S5.

*1.3. Structural Details*

In the following sections, in comparison of energies of different tautomers and conformers, only data from calculations of BP86/def-TZVP basis set are used because other methods were not used to calculate the less stable forms. *Relative Gibbs free energy values* (ΔG) in the gas-phase are provided to compare the stability of different tautomers and conformers. The reader must note that it is not the GA value.

1.3.1. Central atom O

Divalent oxygen substituted with the CN group exhibits two tautomers: OH-acid (cyanic acid, **1H-T1**) and NH-acid (isocyanic acid **1H-T2**, Figure 7 in the main text). The replacement of the CN group with the TCNV group gives a structure with five possible tautomers (Figure S2). The most stable form is OH-acid, tautomer **T1**.

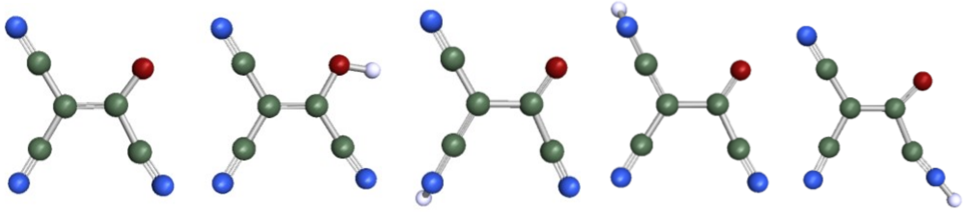

| | **2** | **2H-T1** | **2H-T4** | **2H-T5** | **2H-T6** |
|---|---|---|---|---|---|
| Δ*G* [kcal/mol] | | 0 | 3.4 | 4.0 | 19.7 |

**Figure S2.** Geometries of **2** and its conjugate acids in different tautomeric forms with their Δ*G* values compared to the most stable **2H-T1**.

Anion **2** is planar. Tautomers **T1**, **T4**-**T5** are also planar, except the proton is bent out of the plane. In the case of **T6**, the C-N-H angle is almost straight (177°), but the C-C-N angle, where the proton is attached, is slightly bent (161°). The whole molecule, however, is fully planar.

Tautomers **T4** and **T5** have a similar energy, **T4** is 0.6 kcal/mol more stable. These tautomers are, respectively, 3.4 and 4.0 kcal/mol less stable than the most stable **T1**. The least stable form is **T6**, which is 19.7 kcal/mol less stable than **T1**. Tautomer **T3**, CH-acid, has different conformers since the possibility to rotate about the C-C bond. Two conformers are computationally archivable: *syn*(H) form and *eclipsed*(H) form (Figure S3). *Syn*(H) form is the most stable among CH-acids, 0.2 kcal/mol more stable than *eclipsed*(H) form. *Anti*(H) is not possible to obtain during structure optimization; however, when the torsion angle is frozen (179°) during the optimization, we obtain the structure which is 2.8 kcal/mol less stable than *syn*(H) form (imaginary frequencies are not zero). **T3** *syn*(H) form is 6.8 kcal/mol less stable than the most stable **2H-T1**.

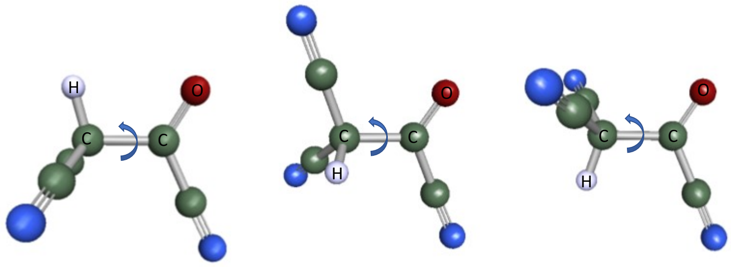


| | **2H-T3** *syn*(H) | **2H-T3** *eclipsed*(H) | **2H-T3** *anti*(H)* |
|---|---|---|---|
| < ω(O-C-C-H) [°] | 1.7 | 117.2 | 179* |
| Δ*G* [kcal/mol] | 6.8 | 7.0 | 9.5 |

**Figure S3.** Three different conformers of compound **2H-T3** and their Δ*G* values compared to the most stable **2H-T1**. **Anti*(H) is not a stable conformer for this compound, having imaginary frequencies. Torsion angles (< ω) of O–C–C–H are presented.

### 1.3.2. Central atom N

The most stable tautomer of dicyanamine has a proton attached to the CN group nitrogen, tautomer **T2** (Figure 7 in the main text).

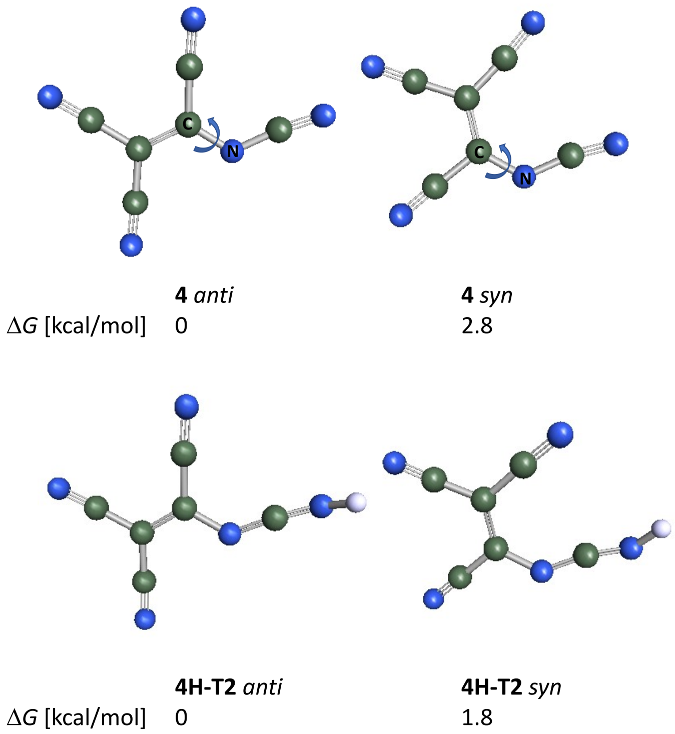


**Figure S4.** Geometry of **4** and its most stable tautomers **4H-T2**.

Its TCNV substituted analog, anion **4,** and its conjugate acids, **4H-T1**, **4H-T2,** and **4H-T6**, can be in *anti* and *syn* conformation. Acids **4H-T3**, **4H-T4**, and **4H-T5** exhibit *trans* and *cis* isomers (Figure S4). *Syn* and *cis* forms are less stable, most probably because of the repulsion between CN and TCNV groups. Anions in *anti* and *syn* conformation are fully planar. Acid forms, except tautomer **T3**, CH-acid, are also planar; only the proton is bent out of the plane. Acid **4H-T3**, differently from **2H-T3**, the *anti*(H) form is also stable; however, the lowest energy is obtained for *syn*(H) conformer (Table S1).

**Table S1.** Δ*G* values (kcal/mol) of the most stable conformers of different tautomers for compound **4H**, relative to the **4H-T2** *anti* conformer at BP86/def-TZVP level.

| **4H-T2** *anti* | **4H-T1** *anti* | **4H-T4** *trans* | **4H-T5** *trans* | **4H-T3** *trans-syn*(H) | **4H-T6** *anti* |
|---|---|---|---|---|---|
| 0 | 6.7 | 15.0 | 15.2 | 18.9 | 32.0 |

The most stable form for anion **5** is the *anti-anti* conformation. The most stable acid form is **T1**, i.e., the proton attached to the central nitrogen atom. Again, three different conformers (*anti-anti*, *anti-syn*, *syn-syn*) for anion and **4H-T1** (Figure S5), and four different conformers/isomers for other protonated forms can be envisaged. Tautomer **T3**, the CH-acid, has again many possibilities to rotate about C-C bond. 8 different conformers are calculated for **T3** tautomer, *trans-anti-syn*(H) is the most stable form (Table S2).

While anion **4** is fully planar molecule, then anion **5** is not planar. Each TCNV group is planar, the C-N-C angles and the angles between two planes formed from each TCNV group's nitrogen atoms, are the higher the more sterically congested the molecule is (Figure 11).

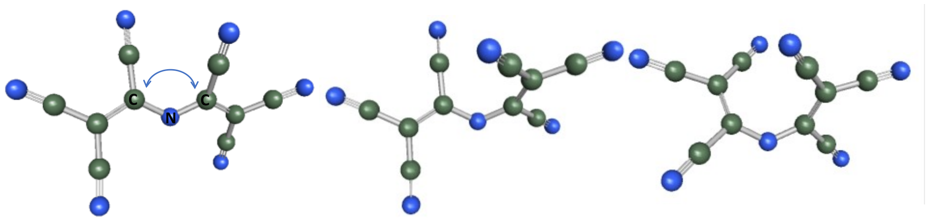


| | **5** *anti-anti* | **5** *anti-syn* | **5** *syn-syn* |
|---|---|---|---|
| < TCNV planes [°] | 45.3 | 57.8 | 62.6 |
| < C-N-C [°] | 125.8 | 126.5 | 129.5 |
| Δ*G* [kcal/mol] | 0 | 1.8 | 4.2 |

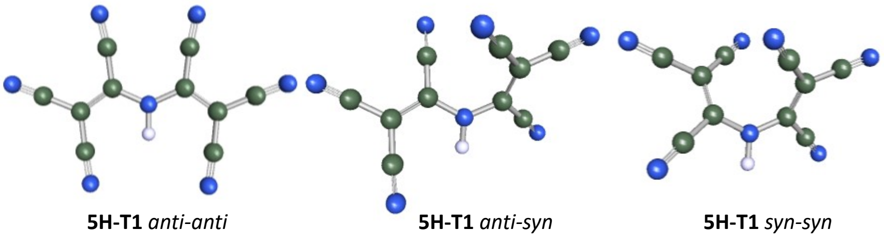

| | **5H-T1** *anti-anti* | **5H-T1** *anti-syn* | **5H-T1** *syn-syn* |
|---|---|---|---|
| < TCNV planes [°] | 4.3 | 53.4 | 51.1 |
| < C-N-C [°] | 131.5 | 127.8 | 130.1 |
| Δ*G* [kcal/mol] | 0 | 1.2 | 3.9 |

**Figure S5.** Geometries of three different conformers of anion **5**. The angle between planes composed with N atoms of TCNV groups and bent between C-N-C atoms are presented. The tautomer **T1** as the most stable acid form with the same parameters are brought.

**Table S2.** Δ*G* values (kcal/mol) of the most stable conformers of different tautomers for compound **5H**, relative to the **5H-T1** *anti-anti* conformer at BP86/def-TZVP level.

| **5H-T1** *anti-anti* | **5H-T5** *trans-anti* | **5H-T4** *trans-anti* | **5H-T3** *trans-anti-syn*(H) | **5H-T6** *anti-anti* |
|---|---|---|---|---|
| 0 | 9.1 | 10.1 | 14.3 | 24.8 |

1.3.3. Central atom C

Replacement of one CN group in cyanoform **6H** with one TCNV group leads to a long-known pentacyanopropene **7H** (Figure S6). The anion **7** and **T6** tautomer of the neutral acid is practically planar (H is bent out of plane 16°). Tautomer **T2** (also **T4** and **T5**) is generally planar, the protonated CN group slightly bent (9–10°) and N-H bond bent out of plane (53°). Tautomer **7H-T3** has stable *syn*(H) and *anti*(H) forms, but the *eclipsed*(H) form is not computationally achievable.

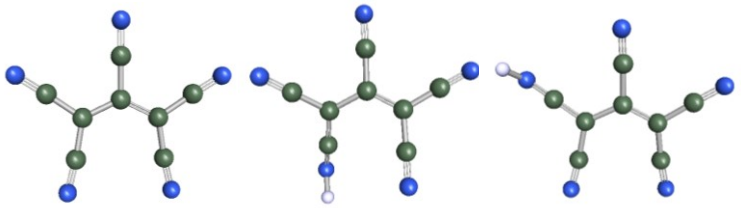

| | **7** | **7H-T2(T5)** | **7H-T2(T4)** |
|---|---|---|---|
| Δ*G* [kcal/mol] | | 0 | 0.5 |

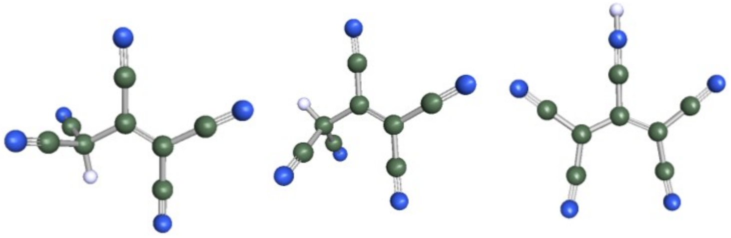

| | **7H-T3** *syn*(H) | **7H-T3** *anti*(H) | **7H-T6** |
|---|---|---|---|
| Δ*G* [kcal/mol] | 2.1 | 4.0 | 17.3 |

**Figure S6.** Geometries of **7** and its conjugate acids with their Δ*G* values with respect to the most stable **T2**(**T5**) tautomer.

Second replacement of CN group with TCNV group leads to an anion **8** (Figure S7). Similarly, to N-centered **5**, placing CN group downwards, *syn-syn*, *syn-anti*, and *anti-anti* conformers can be envisaged. Differently from **5**, not an *anti-anti* conformer but the *anti-syn* conformer has the lowest energy while the *anti-anti* form is the least stable one, however, the differences are minor. The torsion angle between TCNV groups (measured as an angle between the planes composed of N atom of TCNV group, < TCNV group) is not consistent with the stability. **8H-T2** tautomer with *anti-syn* conformation is the most stable acid form. The plane using three C atoms around central atom C has been composed and the distance of the central atom C from the plane has been measured (d C-CCC plane), which shows the planarity of the central part of the structure. We can see that the central part is practically planar.

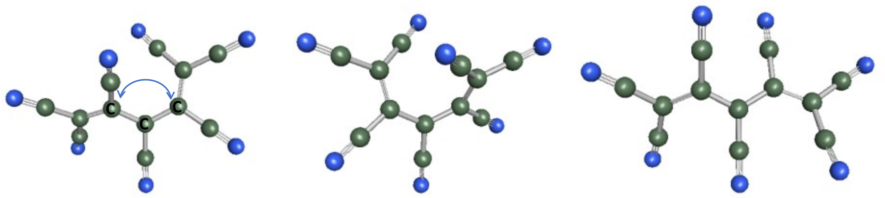


| | **8** *anti-syn* | **8** *syn-syn* | **8** *anti-anti* |
|---|---|---|---|
| < TCNV planes [°] | 58.8 | 44.7 | 63.2 |
| < C-C-C [°] | 123.4 | 126.7 | 122.9 |
| d C – CCC plane [Å] | 0.04 | 0.00 | 0.00 |
| Δ*G* [kcal/mol] | 0 | 0.6 | 0.7 |

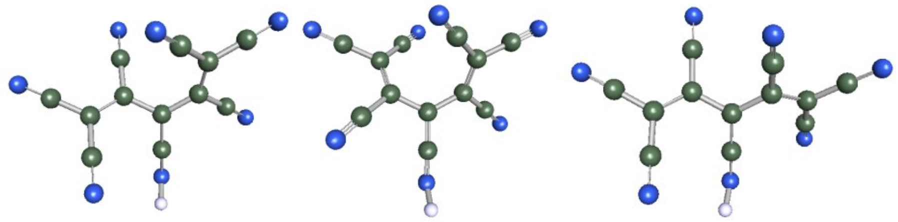

| | **8H-T2** *anti-syn* | **8H-T2** *syn-syn* | **8H-T2** *anti-anti* |
|---|---|---|---|
| < TCNV planes [°] | 61.0 | 56.3 | 69.6 |
| < C-C-C [°] | 123.8 | 126.5 | 121.4 |
| d C – CCC plane [Å] | 0.03 | 0.01 | 0.02 |
| Δ*G* [kcal/mol] | 0 | 1.9 | 0.1 |

**Figure S7.** Different conformers of anion **8** and acid form **8H-T2**.

In Table S3 the stability of tautomers of the most stable conformers are available. The differences between different tautomers become smaller compared to the above presented compounds the more TCNV groups the compound incorporates.

**Table S3.** Δ*G* values (kcal/mol) of the most stable conformers of different tautomers for compound **8H**, relative to the **8H-T2** *anti-syn* conformer at BP86/def-TZVP level.

| **8H-T2** *anti-syn* | **8H-T5** *trans-syn* | **8H-T4** *trans-syn* | **8H-T1** *anti-syn* | **8H-T3** *trans-syn-syn*(H) | **8H-T6** *anti-syn* |
|---|---|---|---|---|---|
| 0 | 1.5 | 2.6 | 4.0 | 4.4 | 16.2 |

The third replacement of CN group by TCNV group gives the compound **9** with altogether 9 CN groups (Figure S8). In the case of all C-centered anions, also in this case, the central atom C is practically on the same plane as its neighboring C-atoms, therefore, two different anions can be envisaged, the lower energy symmetrical and 2.0 kcal/mol higher energy asymmetrical.

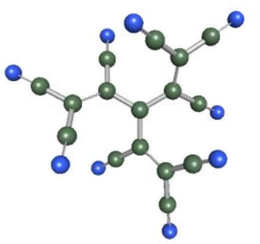

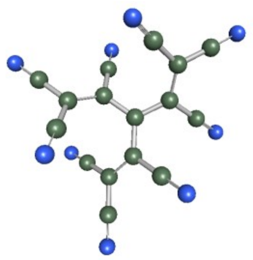

| | **9** *symmetrical* | **9** *asymmetrical* |
|---|---|---|
| < C-C-C [°] | 119.9 | 118.7 |
| | 119.9 | 118.8 |
| | 119.9 | 122.6 |
| d C – CCC plane [Å] | 0.05 | 0.02 |
| Δ*G* [kcal/mol] | 0 | 2.0 |

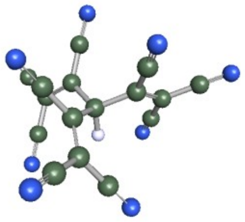

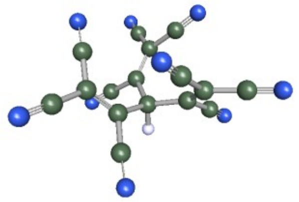

| | **9H-T1** *anti-anti-anti* | **9H-T1** *syn-syn-syn* |
|---|---|---|
| < C-C-C [°] | 112.0 | 115.4 |
| | 112.1 | 115.5 |
| | 111.9 | 115.5 |
| d C – CCC plane [Å] | 0.44 | 0.33 |
| Δ*G* [kcal/mol] | 0 | 8.3 |

**Figure S8.** Anions **9** with its two conformers and the most stable conjugate acid forms **9H-T1**.

The most stable acid form, **T1**, is slightly more tetrahedron-like, with smaller C-C angle and seemingly higher distance of the central atom from the plane composed its neighbouring C-atoms. The stability of different tautomers shows that **T1, T4** and **T5** tautomers have similar energies (Table S4) and very close GA values (see Table S9). **T6** tautomer comes also energetically closer to other tautomers.

**Table S4.** Δ*G* values (kcal/mol) of the most stable conformers of different tautomers for compound **9H**, relative to the **9H-T1** *anti-anti-anti* conformer at BP86/def-TZVP level.

| **9H-T1** *symmetrical anti-anti-anti* | **9H-T4** *symmetrical trans-anti-anti* | **9H-T5** *symmetrical trans-anti-anti* | **9H-T3** *symmetrical trans-anti-anti- syn*(H) | **9H-T6** *symmetrical anti-anti-anti* |
|---|---|---|---|---|
| 0 | 1.7 | 3.0 | 7.9 | 13.3 |

1.3.4. Central atom B

**T1** and **T3** tautomers of any boron containing compounds were not achievable computationally, resulting trivalent boron compounds.

Tetracyanoborate, a tetrahedral anion (Figure 7 in the main text), can be replaced with up to four TCNV groups while the configuration stays close to tetrahedron. The angles between bulky TCNV groups, depending on their conformation, are generally somewhat higher compared to the angles between CN groups.

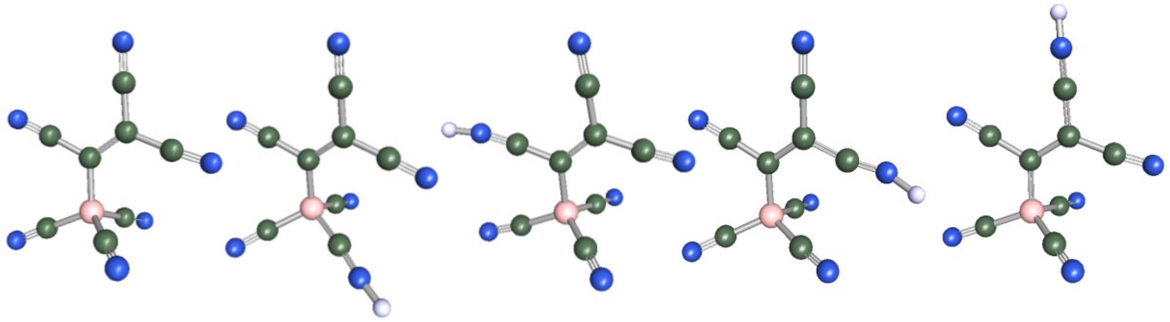

| | **11** | **11H-T2** | **11H-T6** | **11H-T5** | **11H-T4** |
|---|---|---|---|---|---|
| Δ*G* [kcal/mol] | | 0 | 11.2 | 13.2 | 19.5 |

**Figure S9.** Anion **11** and its different tautomers of conjugate acids.

The most stable tautomer of acid **11H** (Figure S9) is the **T2** tautomer. Three different **T2** tautomers were calculated for each conformation. Differently from O-, N- and C-centered acids, **T6** tautomer is more stable than **T4** and **T5** tauromers.

The second replacement of the CN group with the TCNV group leads to an anion (**12**) where three different conformers can be envisaged (Figure S10). Differently from **5**, where *anti-anti*, and from **8**, where *anti-syn* were the most stable conformers, in the case of **12**, most probably because of an additional repulsion from CN groups, the *syn-syn* conformer is the most stable form. Very close is the *anti-syn* conformer in its stability. Two different **T2** tautomers were calculated for each anion, whereas the *anti-syn* form gave the lowest GA value because its protonated form has the highest stability. Again, as shown in Figure 5 in the main text and Table S5, **T6** tautomers are close to **T5** tautomers in their stability. To compare the structures with anions whose central atoms are N (**5**) and C (**8**) atoms, we can see that the C-X-C angle has the smallest value. This comes from the tetrahedral configuration of borates. For anions **5** and **8**, these angles have very similar values being in the range of 123–130 degrees, being the smallest for *anti-anti* conformers and the largest for *syn-syn* conformers.

**Table S5.** Δ*G* values (kcal/mol) of the most stable conformers of different tautomers for compound **12H**, relative to the **12H-T2** *anti-syn* conformer at BP86/def-TZVP level.

| **12H-T2** *anti-syn* | **12H-T6** *syn-anti* | **12H-T5** *trans-syn* | **12H-T4** *trans-syn* |
|---|---|---|---|
| 0 | 10.5 | 13.2 | 17.6 |

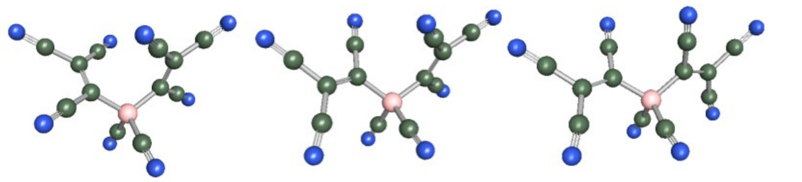

| | **12** *syn-syn* | **12** *anti-syn* | **12** *anti-anti* |
|---|---|---|---|
| < TCNV planes [°] | 77.1 | 69.9 | 65.5 |
| < C-B-C [°] | 113.3 | 111.0 | 109.3 |
| Δ*G* [kcal/mol] | 0 | 0.1 | 0.8 |

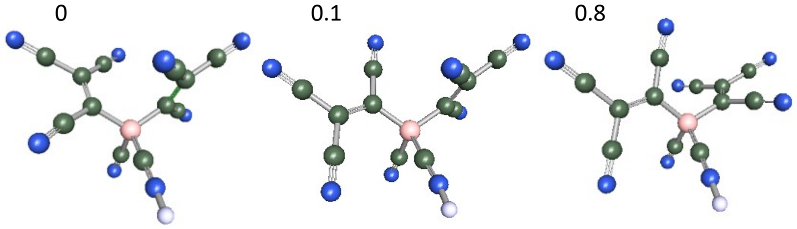

| | **12H-T2** *syn-syn* | **12H-T2** *anti-syn* | **12H-T2** *anti-anti* |
|---|---|---|---|
| < TCNV planes [°] | 82.0 | 78.5 | 71.1 |
| < C-B-C [°] | 115.9 | 112.4 | 113.3 |
| Δ*G* [kcal/mol] | 1.1 | 0 | 1.1 |

**Figure S10.** Three conformers of anion **12** and acid **12H-T2** with their Δ*G* values. The angle between TCNV planes and C-B-C atoms is indicated.

The third replacement of the CN group with the TCNV group gives large-sized anion **13** with four different conformers. Their stability is not too different; however, the most congested *syn-syn-syn* form is the least stable. *Anti-anti-anti* **13H-T2** is the most stable acid form, leaving the second stable **T6** tautomer behind more than 10 kcal/mol (Table S6).

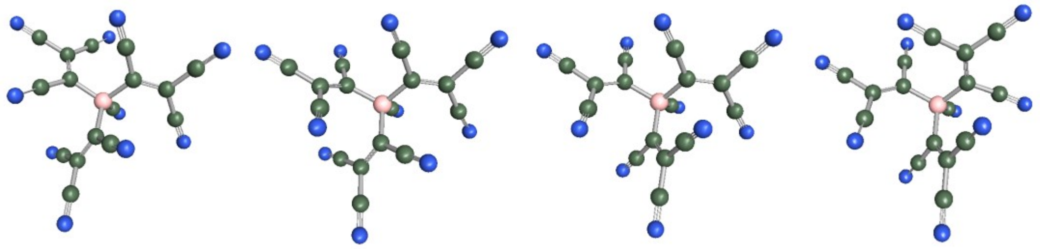

| | **13** *anti-anti-anti symmetrical* | **13** *anti-anti-syn* | **13** *anti-syn-syn* | **13** *syn-syn-syn symmetrical* |
|---|---|---|---|---|
| < C-B-C [°] | 109.9 | 108.8 | 109.0 | 113.2 |
| | 109.9 | 111.3 | 112.0 | 113.4 |
| | 109.9 | 112.4 | 115.1 | 113.4 |
| Δ*G* [kcal/mol] | 0 | 0.9 | 1.5 | 2.7 |

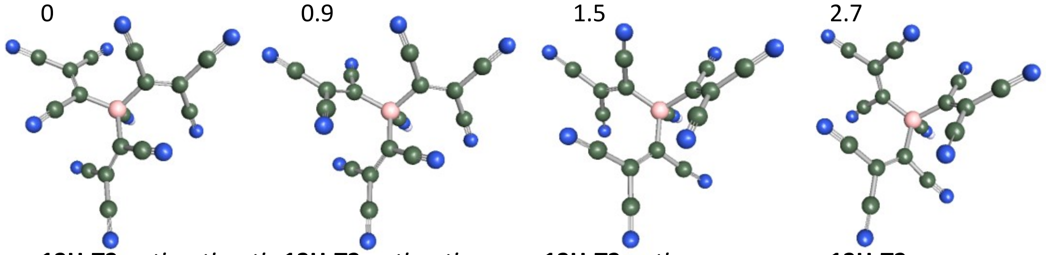

| | **13H-T2** *anti-anti-anti symmetrical* | **13H-T2** *anti-anti-syn* | **13H-T2** *anti-syn-syn* | **13H-T2** *syn-syn-syn symmetrical* |
|---|---|---|---|---|
| < C-B-C [°] | 111.1 | 114.7 | 110.2 | 115.2 |
| | 111.4 | 112.3 | 113.4 | 115.1 |
| | 111.5 | 110.4 | 117.0 | 115.0 |
| Δ*G* [kcal/mol] | 0 | 0.5 | 1.1 | 0.9 |

**Figure S11.** Different conformers of anion **13** and its Δ*G* values compared to the most stable, the least congested symmetrical *anti-anti-anti* conformer.

**Table S6.** Δ*G* values (kcal/mol) of the most stable conformers of different tautomers for compound **13H**, relative to the **13H-T2** *anti-anti-anti* conformer at BP86/def-TZVP level.

| **13H-T2** *anti-anti-anti* | **13H-T5** *trans-anti-anti* | **13H-T6** *anti-anti-anti* | **13H-T4** *trans-anti-anti* |
|---|---|---|---|
| 0 | 13.0 | 14.3 | 19.5 |

The fourth replacement of the CN group with the TCNV group is the most interesting (Figure S12). The most basic site, a single CN group, is now missing. Since no specific configurations for anion could be envisaged, the program COSMOconf was used to find the most stable conformers for anion. Then, four of the most stable anion conformers were chosen, and further calculations were carried out with these. GA values for all the protonation sites in the case of **T4**, **T5,** and **T6** tautomers were calculated. The most stable form is, interestingly, the **T6** tautomer (Table S7).

**Table S7.** Δ*G* values (kcal/mol) of the most stable conformers of different tautomers for compound **14H**, relative to the **14H-T6** conformer at BP86/def-TZVP level.

| **14H-T6** | **14H-T5** | **14H-T4** |
|---|---|---|
| 0 | 4.0 | 4.7 |

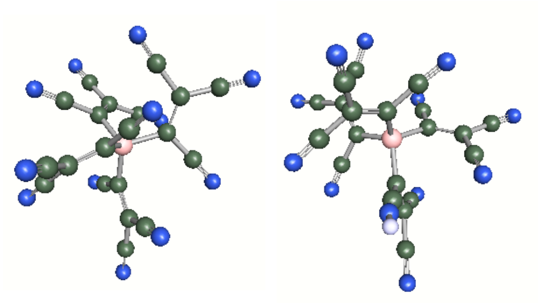

| | **14** | **14H-T6** |
|---|---|---|
| < C-B-C [°] | 109.2 | 108.2 |
| | 109.2 | 110.3 |
| | 109.6 | 110.4 |
| | 109.7 | 111.4 |
| < H-N-C [°] | | 172.1 |

**Figure S12.** The most stable conformers of anion **14** and its most stable acid form, tautomer **T6**.

1.3.5. Additional acids

Anions of $CN^-$ (**15**) and $TCNV^-$ (**16**) were included in this work for comparison. HCN and HNC are stick-like, and the proton is not bent. Protonated TCNV anion forms interesting structures during optimization (Figure S13); different tautomers exhibit huge instability compared to the most stable acid form.

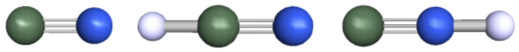

| | 15 | 15H-T1 | 15H-T2 |
|---|---|---|---|
| Δ*G* [kcal/mol] | | 0 | 14.1 |

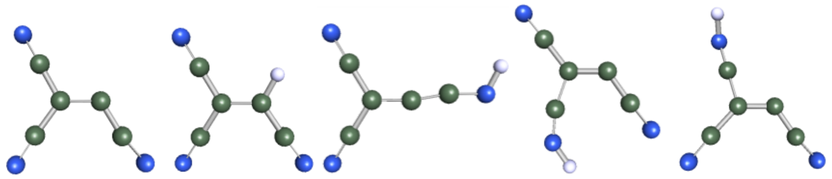

| | 16 | 16H-T1 | 16H-T6 | 16H-T4 | 16H-T5 |
|---|---|---|---|---|---|
| Δ*G* [kcal/mol] | | 0 | 26.4 | 50.1 | 53.0 |

**Figure S13.** Geometries and Δ*G* values of $CN^-$ and $TCNV^-$ anions and their acid forms.

Champions in acidity, carborane and dodecaborate acids were included in this work. Only the fully cyanated molecules were studied. As seen from Figure S14, the most stable neutral form of dodecaborate is the one where both protons are as far as possible from each other. The most stable neutral form for carborane acid is the proton attached to the CN group utmost the C-atom of the carborane cage (12 vertex).

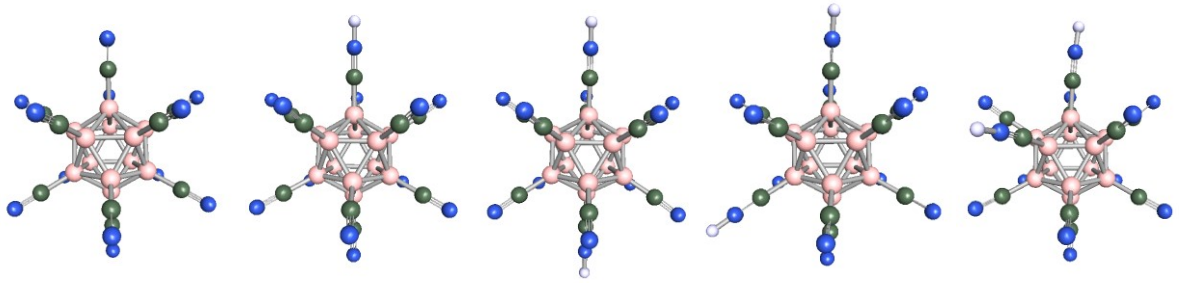

| | 17 | 18 | 18-1 | 18-2 | 18-3 |
|---|---|---|---|---|---|
| | *dianion* | *anion* | *neutral* | *neutral* | *neutral* |
| Δ*G* [kcal/mol] | | | 0 | 1.7 | 9.0 |

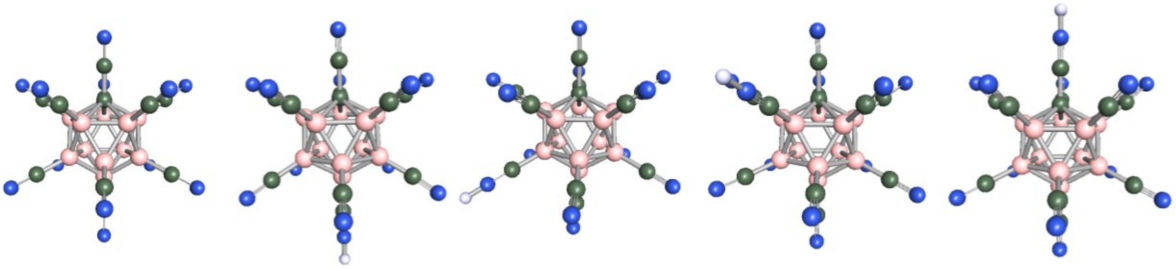

| | 19 | 19-1 | 19-2 | 19-3 | 19-4 |
|---|---|---|---|---|---|
| | *anion* | *neutral* | *neutral* | *neutral* | *neutral* |
| Δ*G* [kcal/mol] | | 0 | 2.2 | 7.6 | 15.0 |

**Figure S14.** Geometries of dodecaborate dianion **17** and carborane **19** and their protonated forms.

**Table S8.** p$K_a$ values in different solvents used for final results.

| | Compound | COSMO-RS p$K_a$(MeCN) not corrected | COSMO-RS p$K_a$(MeCN) corrected | COSMO-RS p$K_a$(DMSO) | p$K_a$(DMSO) correlation | p$K_a$(DMSO) correlation | AVERAGE p$K_a$(DMSO) | COSMO-RS p$K_a$($H_2O$) | p$K_a$(DCE) correlation | p$K_a$(DCE) absolute |
|---|---|---|---|---|---|---|---|---|---|---|
| | | **A** | **B** | **C** | **D** | **E** | **F** | **G** | **H** | **I** |
| **1H** | [CN-O]H | 22.0 | 24.2 | 10.6 | 12.1 | 12.8 | 12.4 | 4.8 | 14.7 | 59.7 |
| **2H** | [O-TCNV]H | 2.9 | 5.1 | -5.1 | -7.4 | -6.7 | -7.0 | -3.2 | -6.6 | 38.4 |
| **3H** | $[(CN)_2N]H$ | 10.3 | 12.5 | 0.9 | 0.2 | 0.9 | 0.5 | 0.0 | 1.7 | 46.7 |
| **4H** | [CN-N-TCNV]H | 3.6 | 5.7 | -4.9 | -6.7 | -6.0 | -6.3 | -2.7 | -5.8 | 39.2 |
| **5H** | $[N\text{-}TCNV_2]H$ | -1.0 | 1.2 | -8.6 | -11.4 | -10.6 | -11.0 | -4.5 | -10.9 | 34.1 |
| **6H** | $[(CN)_3C]H$ | 2.8 | 4.9 | -5.4 | -7.6 | -6.8 | -7.2 | -3.6 | -6.8 | 38.2 |
| **7H** | $[(CN)_2\text{-}C\text{-}TCNV]H$ | -4.6 | -2.4 | -11.2 | -15.1 | -14.3 | -14.7 | -7.3 | -15.0 | 30.0 |
| **8H** | $[CN\text{-}C\text{-}TCNV_2]H$ | -7.3 | -5.2 | -13.3 | -17.9 | -17.1 | -17.5 | -8.7 | -18.0 | 27.0 |
| **9H** | $[C\text{-}TCNV_3]H$ | -9.7 | -7.6 | -15.0 | -20.3 | -19.6 | -20.0 | -10.0 | -20.7 | 24.3 |
| **10H** | $[(CN)_4B]H$ | -5.6 | -3.5 | -11.5 | -16.1 | -15.4 | -15.8 | -7.8 | -16.1 | 28.9 |
| **11H** | $[(CN)_3\text{-}B\text{-}TCNV]H$ | -6.2 | -4.1 | -12.0 | -16.8 | -16.0 | -16.4 | -8.0 | -16.8 | 28.2 |
| **12H** | $[(CN)_2\text{-}B\text{-}TCNV_2]H$ | -7.1 | -4.9 | -12.6 | -17.6 | -16.9 | -17.2 | -8.3 | -17.7 | 27.3 |
| **13H** | $[CN\text{-}B\text{-}TCNV_3]H$ | -8.0 | -5.9 | -13.3 | -18.6 | -17.9 | -18.2 | -8.8 | -18.8 | 26.2 |
| **14H** | $[B\text{-}TCNV_4]H$ | -15.0 | -12.9 | -18.6 | -25.7 | -25.0 | -25.3 | -13.0 | -26.6 | 18.4 |
| **15H** | HCN | 26.2 | 28.4 | 14.6 | 16.4 | 17.1 | 16.8 | 8.6 | 19.4 | 64.4 |
| **16H** | $C(CN)_2{=}CH(CN)$ | 27.4 | 29.7 | 13.2 | 17.7 | 18.4 | 18.1 | 11.6 | 20.8 | 65.8 |
| **17H** | $[B_{12}(CN)_{12}]H^-$ | -10.2 | -8.1 | -14.9 | -20.8 | -20.1 | -20.4 | -11.3 | -21.2 | 23.8 |
| **18H** | $[B_{12}(CN)_{12}]H_2$ | -13.5 | -11.4 | -17.8 | -24.1 | -23.4 | -23.8 | -11.8 | -24.9 | 20.1 |
| **19H** | $[CB_{11}(CN)_{12}]H$ | -16.3 | -14.2 | -19.8 | -27.1 | -26.4 | -26.7 | -13.3 | -28.1 | 16.9 |

**A**: COSMO-RS p$K_a$(MeCN) values obtained from the calculations according to the .cosmo files available in DataDoi data repository. Different tautomers and conformers are taken into account.
**B**: COSMO-RS p$K_a$(MeCN) values that are corrected according to Eq 4 in the main text.
**C**: COSMO-RS p$K_a$(DMSO) values obtained from the calculations according to the .cosmo files available in DataDoi data repository.
**D**: p$K_a$(DMSO) values that are obtained from the correlation from corrected p$K_a$(MeCN) values (column B) using the equation from Ref [42] in the main text.
**E**: p$K_a$(DMSO) values that are obtained from the correlation from corrected p$K_a$(MeCN) values (column B) using the equation from Ref [34] in the main text.
**F**: Final assigned p$K_a$(DMSO) values that are obtained by averaging p$K_a$(DMSO) values from columns D and E.
**G**: COSMO-RS p$K_a$($H_2O$) values obtained from the calculations according to the .cosmo files available in DataDoi data repository. The values are not corrected.
**H**: Relative p$K_a$(DCE) values that are obtained from the correlation from corrected p$K_a$(MeCN) values (column B) using the equation from Ref [3] in the main text.
**G**: Absolute p$K_a$(DCE) values according to the Ref [37] in the main text. All these values are 45.0 units higher than the values in column H.

**Table S9.** The total molecular energies, PA and GA values, and a number of imaginary frequencies (N imag) of the studied molecules. When different conformers are named, the first group in the row of the names is always the protonated TCNV group, and counting continues clockwise, or the position of the proton is specifically noted. A single CN group or proton, in the case of **T1** and **T2**, is generally facing downwards or backward. Conformation of the TCNV group in the case of compounds **9H** and **13H** are determined regarding the C-C-C plane around central atom C or B, respectively; the conformation is decided according to the CN group in 1st position of the TCNV group.

| Compound | Basis set | Total energy Ha | Enthalpy Ha | *H* Ha | PA kcal/mol | Chem. Pot. Ha | *G* Ha | GA kcal/mol | N imag |
|---|---|---|---|---|---|---|---|---|---|
| **1** | | | | | | | | | |
| Anion | | | | | | | | | |
| | BP86/def-TZVP | -168.207622 | 0.014630 | -168.192992 | | -0.010231 | -168.217852 | | 0 |
| | BP86/def2-TZVPP | -168.214437 | 0.014653 | -168.199784 | | -0.010202 | -168.224639 | | 0 |
| | BP86/def2-TZVPPD | -168.218957 | 0.014584 | -168.204373 | | -0.010276 | -168.229233 | | 0 |
| | B3LYP/def2-TZVPPD | -168.122537 | 0.015239 | -168.107298 | | -0.001430 | -168.123967 | | 0 |
| | B3LYP/6-311+G(d,p) | | | -168.172144 | | | -168.197063 | | 0 |
| | G4(MP2) | | | -167.955641 | | | -167.980531 | | 0 |
| | W1RO | | | -168.200611 | | | -168.225527 | | 0 |
| | CBS-APNO | | | -168.113344 | | | -168.138159 | | 0 |
| Neutral | | | | | | | | | |
| T1 | BP86/def-TZVP | -168.716794 | 0.024878 | -168.691916 | 314.6 | -0.002685 | -168.719479 | 308.5 | 0 |
| T2 | BP86/def-TZVP | -168.764542 | 0.024798 | -168.739744 | 344.6 | -0.002388 | -168.766930 | 338.3 | 0 |
| | BP86/def2-TZVPP | -168.774659 | 0.024827 | -168.749832 | 346.6 | -0.002359 | -168.777018 | 340.3 | 0 |
| | BP86/def2-TZVPPD | -168.775643 | 0.024820 | -168.750823 | 344.4 | -0.002365 | -168.778008 | 338.1 | 0 |
| | B3LYP/def2-TZVPPD | -168.678486 | 0.025321 | -168.653165 | 344.0 | -0.001785 | -168.680271 | 342.8 | 0 |
| | B3LYP/6-311+G(d,p) | | | -168.713282 | 341.0 | | -168.740361 | 334.6 | 0 |
| | G4(MP2) | | | -168.497657 | 341.6 | | -168.524743 | 335.2 | 0 |
| | W1RO | | | -168.742541 | 341.5 | | -168.769626 | 335.1 | 0 |
| | CBS-APNO | | | -168.655663 | 341.8 | | -168.682714 | 335.4 | 0 |
| **2** | | | | | | | | | |
| Anion | | | | | | | | | |
| | BP86/def-TZVP | -430.226987 | 0.047818 | -430.179169 | | 0.004879 | -430.222108 | | 0 |
| | BP86/def2-TZVPP | -430.245433 | 0.047779 | -430.197654 | | 0.004868 | -430.240565 | | 0 |
| | BP86/def2-TZVPPD | -430.248071 | 0.047724 | -430.200347 | | 0.004780 | -430.243291 | | 0 |
| | B3LYP/def2-TZVPPD | -429.993634 | 0.049008 | -429.944626 | | 0.006489 | -429.987145 | | 0 |
| | B3LYP/6-311+G(d,p) | | | -430.118532 | | | -430.160900 | | 0 |
| | G4(MP2) | | | -429.535840 | | | -429.578200 | | 0 |
| | W1RO | | | -430.171189 | | | -430.213701 | | 0 |
| | CBS-APNO | | | -429.989995 | | | -430.032120 | | 0 |
| Neutral | | | | | | | | | |

| | | | | | | | | | |
|---|---|---|---|---|---|---|---|---|---|
| T1 | BP86/def-TZVP | -430.698883 | 0.060176 | -430.638707 | 289.8 | 0.016565 | -430.682319 | 282.5 | 0 |
| | BP86/def2-TZVPP | -430.720183 | 0.060251 | -430.659932 | 291.6 | 0.016697 | -430.703486 | 284.2 | 0 |
| | BP86/def2-TZVPPD | -430.721855 | 0.060231 | -430.661624 | 290.9 | 0.016709 | -430.705146 | 283.5 | 0 |
| | B3LYP/def2-TZVPPD | -430.468759 | 0.061838 | -430.406921 | 291.6 | 0.018721 | -430.450038 | 284.2 | 0 |
| | B3LYP/6-311+G(d,p) | | | -430.577397 | 289.4 | | -430.620442 | 282.1 | 0 |
| | G4(MP2) | | | -430.002708 | 294.4 | | -430.045755 | 287.1 | 0 |
| | W1RO | | | -430.637035 | 293.8 | | -430.680197 | 286.5 | 0 |
| | CBS-APNO | | | -430.453922 | 292.6 | | -430.49682 | 285.3 | 0 |
| T3; eclipsed(H) | BP86/def-TZVP | -430.685699 | 0.059358 | -430.626341 | 282.1 | 0.014557 | -430.671142 | 275.5 | 0 |
| T3; syn(H) | BP86/def-TZVP | -430.686088 | 0.059539 | -430.626549 | 282.2 | 0.014566 | -430.671522 | 275.7 | 0 |
| T3; anti(H) | BP86/def-TZVP | -430.683587 | 0.058586 | -430.625001 | 281.2 | 0.016476 | -430.667111 | 273.0 | 1 |
| T4 | BP86/def-TZVP | -430.691937 | 0.059061 | -430.632876 | 286.2 | 0.015090 | -430.676847 | 279.1 | 0 |
| T5 | BP86/def-TZVP | -430.691022 | 0.059076 | -430.631946 | 285.6 | 0.015018 | -430.676004 | 278.5 | 0 |
| T6 | BP86/def-TZVP | -430.663974 | 0.057860 | -430.606114 | 269.4 | 0.012976 | -430.650998 | 262.9 | 0 |
| **3** | | | | | | | | | |
| Anion | | | | | | | | | |
| | BP86/def-TZVP | -240.607141 | 0.025478 | -240.581663 | | -0.006791 | -240.613933 | | 0 |
| | BP86/def2-TZVPP | -240.618830 | 0.025484 | -240.593346 | | -0.006740 | -240.625570 | | 0 |
| | BP86/def2-TZVPPD | -240.621254 | 0.025456 | -240.595798 | | -0.006778 | -240.628032 | | 0 |
| | B3LYP/def2-TZVPPD | -240.474743 | 0.026101 | -240.448642 | | -0.005964 | -240.480707 | | 0 |
| | B3LYP/6-311+G(d,p) | | | -240.548045 | | | -240.580080 | | 0 |
| | G4(MP2) | | | -240.216648 | | | -240.248658 | | 0 |
| | W1RO | | | -240.568517 | | | -240.600555 | | 0 |
| | CBS-APNO | | | -240.46843 | | | -240.500308 | | 0 |
| Neutral | | | | | | | | | |
| T1 | BP86/def-TZVP | -241.101085 | 0.037495 | -241.063590 | 303.9 | 0.003874 | -241.097211 | 297.0 | 0 |
| T2 | BP86/def-TZVP | -241.116908 | | -241.116908 | 337.3 | 0.003535 | -241.113374 | 307.1 | 0 |
| | BP86/def2-TZVPP | -241.131063 | 0.036729 | -241.094334 | 315.9 | 0.003631 | -241.127432 | 308.6 | 0 |
| | BP86/def2-TZVPPD | -241.132002 | 0.036724 | -241.095278 | 314.9 | 0.003624 | -241.128378 | 307.7 | 0 |
| | B3LYP/def2-TZVPPD | -240.984167 | 0.037589 | -240.946578 | 313.9 | 0.004685 | -240.979482 | 306.7 | 0 |
| | B3LYP/6-311+G(d,p) | | | -241.042429 | 311.7 | | -241.075302 | 304.5 | 0 |
| | G4(MP2) | | | -240.709896 | 311.0 | | -240.742776 | 303.8 | 0 |
| | W1RO | | | -241.061506 | 310.8 | | -241.094404 | 303.6 | 0 |
| | CBS-APNO | | | -240.961639 | 311.0 | | -240.994359 | 303.7 | 0 |
| **4** | | | | | | | | | |
| Anion | | | | | | | | | |
| anti | BP86/def-TZVP | -502.618281 | 0.060086 | -502.558195 | | 0.012379 | -502.605902 | | 0 |

| | | | | | | | | | |
|---|---|---|---|---|---|---|---|---|---|
| | BP86/def2-TZVPP | -502.641764 | 0.060064 | -502.581700 | | 0.012401 | -502.629363 | | 0 |
| | BP86/def2-TZVPPD | -502.644033 | 0.060001 | -502.584032 | | 0.012292 | -502.631741 | | 0 |
| | B3LYP/def2-TZVPPD | -502.338368 | 0.061590 | -502.276778 | | 0.014455 | -502.323913 | | 0 |
| | B3LYP/6-311+G(d,p) | | | -502.486274 | | | -502.533246 | | 0 |
| | G4(MP2) | | | -501.791892 | | | -501.838878 | | 0 |
| | W1RO | | | -502.532800 | | | -502.579916 | | 0 |
| | CBS-APNO | | | -502.339803 | | | -502.386434 | | 0 |
| syn | BP86/def-TZVP | -502.613592 | 0.060053 | -502.553539 | | 0.012192 | -502.601400 | | 0 |
| | BP86/def2-TZVPP | -502.636604 | 0.060022 | -502.576582 | | 0.012236 | -502.624368 | | 0 |
| Neutral | | | | | | | | | |
| T1; syn | BP86/def-TZVP | -503.077630 | 0.072579 | -503.005051 | 284.8 | 0.023085 | -503.054544 | 278.1 | 0 |
| T1; anti | BP86/def-TZVP | -503.083250 | 0.072541 | -503.010709 | 285.4 | 0.023885 | -503.059364 | 278.3 | 0 |
| T2; anti | BP86/def-TZVP | -503.092361 | 0.071365 | -503.020996 | 291.9 | 0.022293 | -503.070068 | 285.0 | 0 |
| | BP86/def2-TZVPP | -503.118123 | 0.071368 | -503.046755 | 293.3 | 0.022373 | -503.095750 | 286.4 | 0 |
| | BP86/def2-TZVPPD | -503.119703 | 0.071330 | -503.048373 | 292.9 | 0.022281 | -503.097422 | 285.9 | 0 |
| | B3LYP/def2-TZVPPD | -502.813497 | 0.073115 | -502.740382 | 292.4 | 0.024645 | -502.788852 | 285.5 | 0 |
| | B3LYP/6-311+G(d,p) | | | -502.946737 | 290.4 | | -502.995025 | 283.5 | 0 |
| | G4(MP2) | | | -502.252907 | 290.8 | | -502.301252 | 283.9 | 0 |
| | W1RO | | | -502.993228 | 290.4 | | -503.041704 | 283.5 | 0 |
| | CBS-APNO | | | -502.799239 | 289.8 | | -502.847250 | 282.9 | 0 |
| T2; syn | BP86/def-TZVP | -503.090845 | 0.071325 | -503.019520 | 293.9 | 0.022419 | -503.068426 | 286.8 | 0 |
| | BP86/def2-TZVPP | -503.116373 | 0.071347 | -503.045026 | 295.4 | 0.022545 | -503.093828 | 288.3 | 0 |
| T3; trans, eclipsed(H) | BP86/def-TZVP | -503.061094 | 0.071521 | -502.989573 | 272.2 | 0.022088 | -503.039006 | 265.5 | 0 |
| T3; trans, syn(H) | BP86/def-TZVP | -503.062100 | 0.071635 | -502.990465 | 272.7 | 0.022163 | -503.039937 | 266.1 | 0 |
| T3; cis, anti(H) | BP86/def-TZVP | -503.057950 | 0.071583 | -502.986367 | 273.1 | 0.022316 | -503.035634 | 266.2 | 0 |
| T3; cis, syn(H) | BP86/def-TZVP | -503.060834 | 0.071613 | -502.989221 | 274.9 | 0.022209 | -503.038625 | 268.1 | 0 |
| T4; trans | BP86/def-TZVP | -503.068630 | 0.071146 | -502.997484 | 277.1 | 0.022537 | -503.046093 | 269.9 | 0 |
| T4; cis | BP86/def-TZVP | -503.064892 | 0.071039 | -502.993853 | 277.8 | 0.022164 | -503.042729 | 270.7 | 0 |
| T5; trans | BP86/def-TZVP | -503.068128 | 0.071142 | -502.996986 | 276.8 | 0.022344 | -503.045784 | 269.8 | 0 |
| T5; cis | BP86/def-TZVP | -503.067114 | 0.071113 | -502.996001 | 279.1 | 0.022738 | -503.044376 | 271.7 | 0 |
| T6; anti | BP86/def-TZVP | -503.039063 | 0.070006 | -502.969057 | 259.3 | 0.019915 | -503.019148 | 253.0 | 0 |
| T6; syn | BP86/def-TZVP | -503.034477 | 0.070179 | -502.964298 | 259.2 | 0.019713 | -503.014764 | 253.1 | 0 |
| | | | | | | | | | |
| **5** | | | | | | | | | |
| Anion | | | | | | | | | |
| Anion; anti-syn (syn facing forward) | BP86/def-TZVP | -764.601870 | 0.094724 | -764.507146 | | 0.03208581 | -764.569784 | | 0 |
| Anion; anti-syn (syn facing backward) | BP86/def-TZVP | -764.601863 | 0.094718 | -764.507145 | | 0.03207300 | -764.569790 | | 0 |
| | BP86/def2-TZVPP | -764.636463 | 0.094656 | -764.541807 | | 0.03207300 | -764.604390 | | 0 |
| anti-anti | BP86/def-TZVP | -764.605066 | 0.094637 | -764.510429 | | 0.03244700 | -764.572619 | | 0 |

| | | | | | | | | | |
|---|---|---|---|---|---|---|---|---|---|
| | BP86/def2-TZVPP | -764.639837 | 0.094581 | -764.545256 | | 0.03166500 | -764.608172 | | 0 |
| | BP86/def2-TZVPPD | -764.642582 | 0.094465 | -764.548117 | | 0.03100900 | -764.611573 | | 0 |
| | B3LYP/def2-TZVPPD | -764.177664 | 0.097000 | -764.080664 | | 0.03470100 | -764.142963 | | 0 |
| | B3LYP/6-311+G(d,p) | | | -764.400802 | | | -764.462556 | | 0 |
| | G4(MP2) | | | -763.345822 | | | -763.407662 | | 0 |
| | CBS-APNO | | | -764.190708 | | | -764.252022 | | 0 |
| syn-syn | BP86/def-TZVP | -764.598170 | 0.094726 | -764.503444 | | 0.03228400 | -764.565886 | | 0 |
| Neutral | | | | | | | | | |
| T1; anti-syn (syn facing forward) | BP86/def-TZVP | -765.051942 | 0.107391 | -764.944551 | 276.0 | 0.04410500 | -765.007837 | 268.6 | 0 |
| T1; anti-syn (syn facing backward) | BP86/def-TZVP | -765.051928 | 0.107361 | -764.944567 | 276.0 | 0.04404181 | -765.007886 | 268.6 | 0 |
| | BP86/def2-TZVPP | -765.087543 | 0.107302 | -764.980241 | 276.6 | 0.04403000 | -765.043513 | 269.3 | 0 |
| T1; syn-syn | BP86/def-TZVP | -765.048189 | 0.107416 | -764.940773 | 275.9 | 0.04460700 | -765.003582 | 268.4 | 0 |
| T1; anti-anti | BP86/def-TZVP | -765.056284 | 0.107464 | -764.948820 | 276.6 | 0.04369700 | -765.012587 | 269.8 | 0 |
| | BP86/def2-TZVPP | -765.092233 | 0.107387 | -764.984846 | 277.3 | 0.04358200 | -765.048651 | 270.1 | 0 |
| | BP86/def2-TZVPPD | -765.094552 | 0.107236 | -764.987316 | 277.1 | 0.04227200 | -765.052280 | 270.3 | 0 |
| | B3LYP/def2-TZVPPD | -764.631166 | 0.110206 | -764.520960 | 277.8 | 0.04687100 | -764.584295 | 270.7 | 0 |
| | B3LYP/6-311+G(d,p) | | | -764.838918 | 276.4 | | -764.901472 | 269.1 | 0 |
| | G4(MP2) | | | -763.792555 | 281.8 | | -763.855306 | 274.6 | 0 |
| | CBS-APNO | | | -764.63221 | 278.5 | | -764.694763 | 271.5 | 0 |
| T3; cis-anti, eclipsed(H) | BP86/def-TZVP | -765.025965 | 0.106032 | -764.919933 | 260.5 | 0.04185933 | -764.984105 | 253.7 | 0 |
| T3; cis-anti, syn(H) | BP86/def-TZVP | -765.025564 | 0.106185 | -764.919379 | 260.2 | 0.04065200 | -764.984912 | 254.2 | 0 |
| T3; trans-anti, syn(H) | BP86/def-TZVP | -765.030874 | 0.106238 | -764.924636 | 261.4 | 0.04114708 | -764.989727 | 255.5 | 0 |
| T3; trans-anti, eclipsed(H) | BP86/def-TZVP | -765.029303 | 0.106140 | -764.923163 | 260.5 | 0.04102600 | -764.988277 | 254.6 | 0 |
| T3; cis-syn, eclipsed(H) | BP86/def-TZVP | -765.025547 | 0.106099 | -764.919448 | 262.5 | 0.04274000 | -764.982807 | 255.3 | 0 |
| T3; cis-syn, syn(H) | BP86/def-TZVP | -765.023788 | 0.106339 | -764.917449 | 261.3 | 0.04118100 | -764.982607 | 255.2 | 0 |
| T3; trans-syn, syn(H) | BP86/def-TZVP | -765.028440 | 0.106418 | -764.922022 | 261.8 | 0.04126600 | -764.987174 | 255.6 | 0 |
| T3; trans-syn, eclipsed(H) | BP86/def-TZVP | -765.029077 | 0.106315 | -764.922762 | 262.3 | 0.04156800 | -764.987509 | 255.8 | 0 |
| T4; cis-anti | BP86/def-TZVP | -765.034108 | 0.105774 | -764.928334 | 265.8 | 0.04181363 | -764.992295 | 258.9 | 0 |
| T4; trans-anti | BP86/def-TZVP | -765.038000 | 0.105731 | -764.932269 | 266.2 | 0.04156986 | -764.996430 | 259.7 | 0 |
| T4; cis-syn | BP86/def-TZVP | -765.033990 | 0.105858 | -764.928132 | 268.0 | 0.04209200 | -764.991898 | 261.0 | 0 |
| T4; trans-syn | BP86/def-TZVP | -765.037230 | 0.105928 | -764.931302 | 267.6 | 0.04198600 | -764.995244 | 260.7 | 0 |
| T5; trans-anti | BP86/def-TZVP | -765.039415 | 0.105696 | -764.933719 | 267.1 | 0.04126900 | -764.998146 | 260.7 | 0 |
| T5; cis-syn | BP86/def-TZVP | -765.030109 | 0.105821 | -764.924288 | 265.6 | 0.04059900 | -764.989510 | 259.5 | 0 |
| T5; trans-syn | BP86/def-TZVP | -765.035593 | 0.105876 | -764.929717 | 266.6 | 0.04164700 | -764.993946 | 259.9 | 0 |
| T5; cis-anti | BP86/def-TZVP | -765.031063 | 0.105717 | -764.925346 | 263.9 | 0.04159800 | -764.989465 | 257.1 | 0 |
| T6; anti-anti | BP86/def-TZVP | -765.012987 | 0.104621 | -764.908366 | 251.2 | 0.03991400 | -764.973073 | 245.0 | 0 |
| T6; syn-syn | BP86/def-TZVP | -765.004797 | 0.104861 | -764.899936 | 250.3 | 0.04024200 | -764.964555 | 243.9 | 0 |
| T6; anti-syn | BP86/def-TZVP | -765.009335 | 0.104766 | -764.904569 | 250.9 | 0.04059800 | -764.968737 | 244.1 | 0 |
| T6; syn-anti | BP86/def-TZVP | -765.007737 | 0.104857 | -764.902880 | 249.8 | 0.03981100 | -764.967926 | 243.6 | 0 |

| | | | | | | | | | |
|---|---|---|---|---|---|---|---|---|---|
| **6** | | | | | | | | | |
| Anion | | | | | | | | | |
| | BP86/def-TZVP | -316.838101 | 0.037123 | -316.800978 | | -0.000673 | -316.838774 | | 0 |
| | BP86/def2-TZVPP | -316.851152 | 0.037055 | -316.814097 | | -0.000747 | -316.851899 | | 0 |
| | BP86/def2-TZVPPD | -316.853112 | 0.037007 | -316.816105 | | -0.000834 | -316.853946 | | 0 |
| | B3LYP/def2-TZVPPD | -316.663083 | 0.037914 | -316.625169 | | 0.000340 | -316.662743 | | 0 |
| | B3LYP/6-311+G(d,p) | | | -316.760517 | | | -316.797325 | | 0 |
| | G4(MP2) | | | -316.317077 | | | -316.353858 | | 0 |
| | W1RO | | | -316.784822 | | | -316.821714 | | 0 |
| | CBS-APNO | | | -316.662846 | | | -316.699558 | | 0 |
| Neutral | | | | | | | | | |
| T1 | BP86/def-TZVP | -317.316257 | 0.048770 | -317.267487 | 294.2 | 0.010067 | -317.306190 | 287.0 | 0 |
| T2 | BP86/def-TZVP | -317.317464 | 0.048394 | -317.269070 | 295.2 | 0.009899 | -317.307564 | 287.9 | 0 |
| | BP86/def2-TZVPP | -317.332801 | 0.048398 | -317.284403 | 296.6 | 0.009906 | -317.322895 | 289.3 | 0 |
| | BP86/def2-TZVPPD | -317.333837 | 0.048380 | -317.285457 | 296.0 | 0.009873 | -317.323964 | 288.7 | 0 |
| | B3LYP/def2-TZVPPD | -317.142341 | 0.049588 | -317.092753 | 294.9 | 0.011412 | -317.130929 | 287.5 | 0 |
| | B3LYP/6-311+G(d,p) | | | -317.225116 | 293.0 | | -317.263178 | 286.0 | 0 |
| | G4(MP2) | | | -316.782681 | 293.6 | | -316.820796 | 286.7 | 0 |
| | W1RO | | | -317.249515 | 293.1 | | -317.287697 | 286.1 | 0 |
| | CBS-APNO | | | -317.127541 | 293.1 | | -317.165395 | 286.0 | 0 |
| **7** | | | | | | | | | |
| Anion | | | | | | | | | |
| | BP86/def-TZVP | -578.840073 | 0.071969 | -578.768104 | | 0.018458 | -578.821616 | | 0 |
| | BP86/def2-TZVPP | -578.865138 | 0.071856 | -578.793282 | | 0.018561 | -578.846577 | | 0 |
| | BP86/def2-TZVPPD | -578.867407 | 0.071772 | -578.795635 | | 0.017646 | -578.849761 | | 0 |
| | B3LYP/def2-TZVPPD | -578.516890 | 0.073615 | -578.443275 | | 0.020553 | -578.496337 | | 0 |
| | B3LYP/6-311+G(d,p) | | | -578.688752 | | | -578.741177 | | 0 |
| | G4(MP2) | | | -577.885015 | | | -577.937095 | | 0 |
| | CBS-APNO | | | -578.528093 | | | -578.579917 | | 0 |
| Neutral | | | | | | | | | |
| T3 = T1; anti(H) | BP86/def-TZVP | -579.282082 | 0.083653 | -579.198429 | 271.5 | 0.029599 | -579.252483 | 264.1 | 0 |
| T3 = T1; syn(H) | BP86/def-TZVP | -579.284980 | 0.083670 | -579.201310 | 273.3 | 0.029412 | -579.255568 | 266.0 | 0 |
| T2 = T4 | BP86/def-TZVP | -579.287094 | 0.083051 | -579.204043 | 275.0 | 0.028951 | -579.258142 | 267.6 | 0 |
| T2 = T5 | BP86/def-TZVP | -579.288599 | 0.083009 | -579.205590 | 276.0 | 0.029706 | -579.258893 | 268.1 | 0 |
| | BP86/def2-TZVPP | -579.315697 | 0.082957 | -579.232740 | 277.2 | 0.029635 | -579.286062 | 269.5 | 0 |
| | BP86/def2-TZVPPD | -579.317462 | 0.082883 | -579.234579 | 276.9 | 0.029432 | -579.288030 | 268.7 | 0 |
| | B3LYP/def2-TZVPPD | -578.965768 | 0.085023 | -578.880745 | 276.0 | 0.032331 | -578.933437 | 268.0 | 0 |

| | | | | | | | | | |
|---|---|---|---|---|---|---|---|---|---|
| | B3LYP/6-311+G(d,p) | | | -579.123607 | 274.4 | | -579.175982 | 266.6 | 0 |
| | G4(MP2) | | | -578.321248 | 275.2 | | -578.373823 | 267.8 | 0 |
| | CBS-APNO | | | -578.961449 | 273.4 | | -579.013592 | 265.9 | 0 |
| T6 | BP86/def-TZVP | -579.257315 | 0.082131 | -579.175184 | 256.9 | 0.026048 | -579.231267 | 250.8 | 0 |
| **8** | | | | | | | | | |
| Anion | | | | | | | | | |
| syn-syn | BP86/def-TZVP | -840.819797 | 0.106493 | -840.713304 | | 0.040279 | -840.779518 | | 0 |
| anti-syn | BP86/def-TZVP | -840.820188 | 0.106488 | -840.713700 | | 0.039776 | -840.780412 | | 0 |
| | BP86/def2-TZVPP | -840.856841 | 0.106409 | -840.750432 | | 0.039752 | -840.817089 | | 0 |
| | BP86/def2-TZVPPD | -840.859815 | 0.106167 | -840.753648 | | 0.038530 | -840.821285 | | 0 |
| | B3LYP/def2-TZVPPD | -840.348210 | 0.108981 | -840.239229 | | 0.042792 | -840.305418 | | 0 |
| | B3LYP/6-311+G(d,p) | | | -840.595443 | | | -840.660817 | | 0 |
| | G4(MP2) | | | -839.434863 | | | -839.500343 | | 0 |
| | CBS-APNO | | | -840.375867 | | | -840.441015 | | 0 |
| anti-anti | BP86/def-TZVP | -840.818932 | 0.106470 | -840.712462 | | 0.039575 | -840.779357 | | 0 |
| | BP86/def2-TZVPP | -840.855634 | 0.106383 | -840.749251 | | 0.039492 | -840.816142 | | 0 |
| Neutral | | | | | | | | | |
| T1; syn-syn | BP86/def-TZVP | -841.242638 | 0.118320 | -841.124318 | 259.4 | 0.049481 | -841.193157 | 253.3 | 0 |
| T1; anti-anti | BP86/def-TZVP | -841.249249 | 0.118370 | -841.130879 | 264.0 | 0.049039 | -841.200210 | 257.8 | 0 |
| T1; anti-syn = syn-anti | BP86/def-TZVP | -841.247075 | 0.118447 | -841.128628 | 261.8 | 0.049382 | -841.197693 | 255.6 | 0 |
| T2; anti-anti | BP86/def-TZVP | -841.253550 | 0.117618 | -841.135932 | 267.2 | 0.049683 | -841.203867 | 260.1 | 0 |
| | BP86/def2-TZVPP | -841.292416 | 0.117539 | -841.174877 | 268.6 | 0.049657 | -841.242759 | 261.4 | 0 |
| T2; syn-anti | BP86/def-TZVP | -841.253972 | 0.117674 | -841.136298 | 266.7 | 0.049923 | -841.204049 | 259.6 | 0 |
| | BP86/def2-TZVPP | -841.292832 | 0.117608 | -841.175224 | 268.0 | 0.049927 | -841.242905 | 260.9 | 0 |
| | BP86/def2-TZVPPD | -841.295398 | 0.116396 | -841.179002 | 268.4 | 0.050723 | -841.244675 | 259.4 | 1 |
| | B3LYP/def2-TZVPPD | -840.783550 | 0.120449 | -840.663101 | 267.5 | 0.052403 | -840.731147 | 260.9 | 0 |
| | B3LYP/6-311+G(d,p) | | | -841.016524 | 265.7 | | -841.083003 | 258.6 | 0 |
| | G4(MP2) | | | -839.858440 | 267.3 | | -839.924998 | 260.2 | 0 |
| | CBS-APNO | | | -840.794097 | 263.9 | | -840.860383 | 256.9 | 0 |
| T2; syn-syn | BP86/def-TZVP | -841.250505 | 0.117636 | -841.132869 | 263.3 | 0.049471 | -841.201034 | 258.2 | 0 |
| T3; cis-anti, anti(H) | BP86/def-TZVP | -841.240404 | 0.118230 | -841.122174 | 257.8 | 0.049580 | -841.190824 | 251.3 | 0 |
| T3; cis-anti, syn(H) | BP86/def-TZVP | -841.241724 | 0.118223 | -841.123501 | 258.6 | 0.048605 | -841.193119 | 252.7 | 0 |
| T3; cis-anti, eclipsed(H) | BP86/def-TZVP | -841.241562 | 0.118102 | -841.123460 | 258.6 | 0.049473 | -841.192089 | 252.1 | 0 |
| T3; trans-anti, syn(H) | BP86/def-TZVP | -841.245605 | 0.118323 | -841.127282 | 261.8 | 0.048582 | -841.197023 | 255.8 | 0 |
| T3; trans-anti, anti(H) | BP86/def-TZVP | -841.243104 | 0.118339 | -841.124765 | 260.2 | 0.049261 | -841.193843 | 253.8 | 0 |
| T3; cis-syn, eclipsed(H) | BP86/def-TZVP | -841.240368 | 0.118014 | -841.122354 | 258.2 | 0.049750 | -841.190618 | 251.7 | 0 |
| T3; trans-syn, syn(H) | BP86/def-TZVP | -841.245773 | 0.118340 | -841.127433 | 261.1 | 0.048742 | -841.197031 | 255.2 | 0 |
| T3; trans-syn, anti(H) | BP86/def-TZVP | -841.242490 | 0.118339 | -841.124151 | 259.0 | 0.049261 | -841.193229 | 252.8 | 0 |

| | | | | | | | | | |
|---|---|---|---|---|---|---|---|---|---|
| T4; cis-anti | BP86/def-TZVP | -841.249331 | 0.117665 | -841.131666 | 263.8 | 0.049472 | -841.199859 | 256.9 | 0 |
| T4; cis-syn | BP86/def-TZVP | -841.250673 | 0.117681 | -841.132992 | 264.8 | 0.049969 | -841.200704 | 258.0 | 0 |
| T4; trans-syn | BP86/def-TZVP | -841.248912 | 0.117640 | -841.131272 | 263.5 | 0.048967 | -841.199945 | 257.0 | 0 |
| T4; trans-anti | BP86/def-TZVP | -841.248112 | 0.117615 | -841.130497 | 263.8 | 0.048609 | -841.199503 | 257.4 | 0 |
| T5; trans-anti | BP86/def-TZVP | -841.250613 | 0.117564 | -841.133049 | 265.4 | 0.049516 | -841.201097 | 258.4 | 0 |
| T5; cis-syn | BP86/def-TZVP | -841.243476 | 0.117516 | -841.125960 | 260.4 | 0.048344 | -841.195132 | 254.5 | 0 |
| T5; cis-anti | BP86/def-TZVP | -841.247755 | 0.117542 | -841.130213 | 262.8 | 0.049631 | -841.198124 | 255.8 | 0 |
| T5; trans-syn | BP86/def-TZVP | -841.251210 | 0.117606 | -841.133604 | 265.0 | 0.049605 | -841.201605 | 258.0 | 0 |
| T6; anti-anti | BP86/def-TZVP | -841.226314 | 0.116810 | -841.109504 | 250.6 | 0.048300 | -841.178014 | 243.9 | 0 |
| T6; anti-syn | BP86/def-TZVP | -841.226771 | 0.116831 | -841.109940 | 250.1 | 0.048584 | -841.178187 | 243.3 | 0 |
| T6; syn-anti | BP86/def-TZVP | -841.225384 | 0.116778 | -841.108606 | 249.3 | 0.047876 | -841.177508 | 242.9 | 0 |
| T6; syn-syn | BP86/def-TZVP | -841.226830 | 0.116809 | -841.110021 | 250.4 | 0.048811 | -841.178019 | 243.8 | 0 |
| **9** | | | | | | | | | |
| Anion | | | | | | | | | |
| symmetrical | BP86/def-TZVP | -1102.795471 | 0.141117 | -1102.654354 | | 0.060374 | -1102.735097 | | 0 |
| | BP86/def2-TZVPP | -1102.843579 | 0.141008 | -1102.702571 | | 0.060361 | -1102.783218 | | 0 |
| | BP86/def2-TZVPPD | -1102.847365 | 0.137131 | -1102.710234 | | 0.061533 | -1102.785832 | | 3 |
| | B3LYP/def2-TZVPPD | -1102.175386 | 0.142990 | -1102.032396 | | 0.063331 | -1102.112055 | | 1 |
| | B3LYP/6-311+G(d,p) | | | -1102.498180 | | | -1102.577184 | | 0 |
| | G4(MP2) | | | -1100.985095 | | | -1101.064353 | | 0 |
| asymmetrical | BP86/def-TZVP | -1102.792184 | 0.141033 | -1102.651151 | | 0.060328 | -1102.731856 | | 0 |
| | BP86/def2-TZVPP | -1102.840299 | 0.140916 | -1102.699383 | | 0.060307 | -1102.779992 | | 0 |
| Neutral | | | | | | | | | |
| T1; asymmetrical, anti-syn-syn; H facing backward | BP86/def-TZVP | -1103.206239 | 0.153113 | -1103.053126 | 253.7 | 0.070041 | -1103.136198 | 247.4 | 0 |
| T1; symmetrical, syn-syn-syn; H facing backward | BP86/def-TZVP | -1103.201043 | 0.153094 | -1103.047949 | 248.5 | 0.069752 | -1103.131292 | 242.3 | 0 |
| T1; symmetrical, anti-anti-anti; H facing backward | BP86/def-TZVP | -1103.212726 | 0.153173 | -1103.059553 | 255.7 | 0.068203 | -1103.144523 | 250.6 | 0 |
| | BP86/def2-TZVPP | -1103.260230 | 0.152924 | -1103.107306 | 255.5 | 0.067924 | -1103.192306 | 250.4 | 0 |
| | BP86/def2-TZVPPD | -1103.263562 | 0.146096 | -1103.117466 | 255.5 | 0.071837 | -1103.191725 | 248.4 | 5 |
| | B3LYP/def2-TZVPPD | -1102.597219 | 0.153142 | -1102.444077 | 258.3 | 0.076612 | -1102.520607 | 250.1 | 3 |
| | B3LYP/6-311+G(d,p) | | | -1102.906469 | 257.7 | | -1102.989190 | 252.3 | 0 |
| | G4(MP2) | | | -1101.404301 | 264.5 | | -1101.487830 | 259.5 | 0 |
| T1; asymmetrical, anti-anti-syn; H facing backward | BP86/def-TZVP | -1103.209141 | 0.153139 | -1103.056002 | 255.5 | 0.069185 | -1103.139956 | 249.8 | 0 |
| T3; trans-syn-syn, eclipsed(H) | BP86/def-TZVP | -1103.199969 | 0.152604 | -1103.047365 | 248.1 | 0.069687 | -1103.130282 | 241.7 | 0 |
| T3; trans-syn-syn, anti(H) | BP86/def-TZVP | -1103.196756 | 0.152712 | -1103.044044 | 246.0 | 0.069340 | -1103.127416 | 241.9 | 0 |
| T3; trans-syn-syn, syn(H) | BP86/def-TZVP | -1103.198806 | 0.152859 | -1103.045947 | 247.2 | 0.069096 | -1103.129710 | 243.4 | 0 |
| T3; trans-syn-anti, syn(H) | BP86/def-TZVP | -1103.200018 | 0.152818 | -1103.047200 | 250.0 | 0.068820 | -1103.131198 | 244.3 | 0 |
| T3; trans-syn-anti, anti(H) | BP86/def-TZVP | -1103.198490 | 0.152737 | -1103.045753 | 249.1 | 0.070263 | -1103.128227 | 242.4 | 0 |
| T3; trans-syn-anti, eclipsed(H) | BP86/def-TZVP | -1103.200162 | 0.152651 | -1103.047511 | 250.2 | 0.069647 | -1103.130515 | 243.9 | 0 |

| | | | | | | | | | |
|---|---|---|---|---|---|---|---|---|---|
| T3; trans-anti-anti, syn(H) | BP86/def-TZVP | -1103.200024 | 0.152870 | -1103.047154 | 250.0 | 0.068900 | -1103.131124 | 244.3 | 0 |
| T3; trans-anti-anti, anti(H) | BP86/def-TZVP | -1103.200867 | 0.152814 | -1103.048053 | 250.5 | 0.070132 | -1103.130735 | 244.0 | 0 |
| T3; trans-anti-anti, syn(H) (symmetrical) | BP86/def-TZVP | -1103.200881 | 0.152884 | -1103.047997 | 248.5 | 0.069013 | -1103.131868 | 242.7 | 0 |
| T3; trans-syn-anti, syn(H) | BP86/def-TZVP | -1103.199664 | 0.152923 | -1103.046741 | 249.7 | 0.068230 | -1103.131434 | 244.5 | 0 |
| T3; trans-syn-anti, anti(H) | BP86/def-TZVP | -1103.199154 | 0.152818 | -1103.046336 | 249.5 | 0.068959 | -1103.130195 | 243.7 | 0 |
| T3; trans-syn-anti, eclipsed(H) | BP86/def-TZVP | -1103.200466 | 0.152666 | -1103.04780 | 250.4 | 0.069360 | -1103.131106 | 244.3 | 0 |
| T4; trans-anti-syn | BP86/def-TZVP | -1103.209442 | 0.152172 | -1103.057270 | 256.3 | 0.069649 | -1103.139793 | 249.7 | 0 |
| T4; trans-syn-anti | BP86/def-TZVP | -1103.212194 | 0.152239 | -1103.059955 | 258.0 | 0.069886 | -1103.142308 | 251.3 | 0 |
| T4; trans-syn-anti | BP86/def2-TZVPP | -1103.262354 | 0.152148 | -1103.110206 | 259.3 | 0.069891 | -1103.192463 | 252.6 | 0 |
| T4; trans-syn-syn | BP86/def-TZVP | -1103.211510 | 0.152215 | -1103.059295 | 257.6 | 0.070007 | -1103.141503 | 250.8 | 0 |
| T4; trans-anti-anti (symmetrical) | BP86/def-TZVP | -1103.211691 | 0.152234 | -1103.059457 | 255.7 | 0.069871 | -1103.141820 | 248.9 | 0 |
| T5; trans-anti-syn | BP86/def-TZVP | -1103.207395 | 0.152080 | -1103.055315 | 255.1 | 0.069530 | -1103.137865 | 248.5 | 0 |
| T5; trans-syn-anti | BP86/def-TZVP | -1103.206349 | 0.152192 | -1103.054157 | 254.4 | 0.068314 | -1103.138035 | 248.6 | 0 |
| T5; trans-syn-syn | BP86/def-TZVP | -1103.205257 | 0.152111 | -1103.053146 | 253.7 | 0.068835 | -1103.136422 | 247.6 | 0 |
| T5; trans-anti-anti (symmetrical) | BP86/def-TZVP | -1103.209401 | 0.152131 | -1103.05727 | 254.3 | 0.069643 | -1103.139758 | 247.6 | 0 |
| T6; anti-anti-syn | BP86/def-TZVP | -1103.189712 | 0.151375 | -1103.038337 | 244.4 | 0.068950 | -1103.120762 | 237.8 | 0 |
| T6; anti-syn-anti | BP86/def-TZVP | -1103.190252 | 0.151366 | -1103.038886 | 244.8 | 0.068512 | -1103.121740 | 238.4 | 0 |
| T6; anti-syn-syn | BP86/def-TZVP | -1103.190605 | 0.151374 | -1103.039231 | 245.0 | 0.069214 | -1103.121391 | 238.2 | 0 |
| T6; anti-anti-anti (symmetrical) | BP86/def-TZVP | -1103.191634 | 0.151417 | -1103.040217 | 243.6 | 0.068231 | -1103.123403 | 237.4 | 0 |
| **10** | | | | | | | | | |
| Anion | | | | | | | | | |
| Pyramidal | BP86/def-TZVP | -396.545269 | 0.046817 | -396.498452 | | 0.002491 | -396.542778 | | 0 |
| | BP86/def2-TZVPP | -396.560182 | 0.046745 | -396.513437 | | 0.002409 | -396.557773 | | 0 |
| | BP86/def2-TZVPPD | -396.562211 | 0.046729 | -396.515482 | | 0.002381 | -396.559830 | | 0 |
| | B3LYP/def2-TZVPPD | -396.337980 | 0.047854 | -396.290126 | | 0.003954 | -396.334026 | | 0 |
| | B3LYP/6-311+G(d,p) | | | -396.461507 | | | -396.505256 | | 0 |
| | G4(MP2) | | | -395.913365 | | | -395.957143 | | 0 |
| | W1RO | | | -396.503497 | | | -396.547444 | | 0 |
| | CBS-APNO | | | -396.354140 | | | -396.397710 | | 0 |
| Neutral | | | | | | | | | |
| T2 | BP86/def-TZVP | -396.980622 | 0.057684 | -396.922938 | 267.8 | 0.011796 | -396.968826 | 261.1 | 0 |
| | BP86/def2-TZVPP | -396.997238 | 0.057550 | -396.939688 | 269.0 | 0.011550 | -396.985688 | 262.2 | 0 |
| | BP86/def2-TZVPPD | -396.998472 | 0.057531 | -396.940941 | 268.5 | 0.011528 | -396.986944 | 261.7 | 0 |
| | B3LYP/def2-TZVPPD | -396.772719 | 0.059001 | -396.713718 | 267.3 | 0.013657 | -396.759062 | 260.4 | 0 |
| | B3LYP/6-311+G(d,p) | | | -396.882396 | 265.6 | | -396.927541 | 258.7 | 0 |
| | G4(MP2) | | | -396.334209 | 265.6 | | -396.379514 | 258.8 | 0 |
| | W1RO | | | -396.924338 | 265.6 | | -396.969744 | 258.7 | 0 |
| | CBS-APNO | | | -396.775450 | 265.9 | | -396.820224 | 258.9 | 0 |

| | | | | | | | | | |
|---|---|---|---|---|---|---|---|---|---|
| **11** | | | | | | | | | |
| Anion | | | | | | | | | |
| | BP86/def-TZVP | -658.513570 | 0.081425 | -658.432145 | | 0.021771 | -658.491798 | | 0 |
| | BP86/def2-TZVPP | -658.540177 | 0.081328 | -658.458849 | | 0.021683 | -658.518494 | | 0 |
| | BP86/def2-TZVPPD | -658.542757 | 0.081235 | -658.461522 | | 0.020935 | -658.521822 | | 0 |
| | B3LYP/def2-TZVPPD | -658.157888 | 0.083339 | -658.074549 | | 0.024135 | -658.133753 | | 0 |
| | B3LYP/6-311+G(d,p) | | | -658.356677 | | | -658.415158 | | 0 |
| | G4(MP2) | | | -657.449462 | | | -657.508125 | | 0 |
| | CBS-APNO | | | -658.186709 | | | -658.244858 | | 0 |
| T2; protonated on CN-1 | BP86/def-TZVP | -658.940838 | 0.092376 | -658.848462 | 262.7 | 0.031236 | -658.909602 | 255.9 | 0 |
| T2; protonated on CN-2 | BP86/def-TZVP | -658.941348 | 0.092348 | -658.849000 | 263.1 | 0.031355 | -658.909993 | 256.1 | 0 |
| T2; protonated on CN-3 | BP86/def-TZVP | -658.941347 | 0.092350 | -658.848997 | 263.1 | 0.031310 | -658.910037 | 256.2 | 0 |
| | BP86/def2-TZVPP | -658.969658 | 0.092197 | -658.877461 | 264.2 | 0.030977 | -658.938681 | 257.4 | 0 |
| | BP86/def2-TZVPPD | -658.971598 | 0.092124 | -658.879474 | 263.7 | 0.029584 | -658.942014 | 257.4 | 0 |
| | B3LYP/def2-TZVPPD | -658.585050 | 0.094574 | -658.490476 | 262.5 | 0.033781 | -658.551269 | 255.7 | 0 |
| | B3LYP/6-311+G(d,p) | | | -658.769801 | 260.7 | | -658.829731 | 253.9 | 0 |
| | G4(MP2) | | | -657.862713 | 260.8 | | -657.923005 | 254.1 | 0 |
| | CBS-APNO | | | -658.599433 | 260.5 | | -658.658830 | 253.5 | 0 |
| T4 | BP86/def-TZVP | -658.908394 | 0.091647 | -658.816747 | 242.8 | 0.029403 | -658.878991 | 236.7 | 0 |
| T5 | BP86/def-TZVP | -658.919180 | 0.091892 | -658.827288 | 249.4 | 0.030224 | -658.888956 | 242.9 | 0 |
| T6 | BP86/def-TZVP | -658.922111 | 0.091737 | -658.830374 | 251.4 | 0.029857 | -658.892254 | 245.0 | 0 |

**12**

Part of the name in () indicates that this group weakly exhibits this conformation.

| | | | | | | | | | |
|---|---|---|---|---|---|---|---|---|---|
| Anion | | | | | | | | | |
| anti-syn; asymmetric | BP86/def-TZVP | -920.479451 | 0.116046 | -920.363405 | | 0.041524 | -920.437927 | | 0 |
| | BP86/def2-TZVPP | -920.517651 | 0.115928 | -920.401723 | | 0.041507 | -920.476144 | | 0 |
| | BP86/def2-TZVPPD | -920.520919 | 0.114788 | -920.406131 | | 0.043038 | -920.477881 | | 1 |
| | B3LYP/def2-TZVPPD | -919.975370 | 0.117884 | -919.857486 | | 0.047633 | -919.927737 | | 1 |
| | B3LYP/6-311+G(d,p) | | | -920.249602 | | | -920.322557 | | 0 |
| | G4(MP2) | | | -918.985830 | | | -919.059162 | | 0 |
| syn-syn; symmetric | BP86/def-TZVP | -920.480079 | 0.116042 | -920.364037 | | 0.041996 | -920.438082 | | 0 |
| anti-anti; symmetric | BP86/def-TZVP | -920.477709 | 0.116039 | -920.361670 | | 0.040930 | -920.436779 | | 0 |
| Neutral | | | | | | | | | |
| T2; protonated on CN-1; anti-syn | BP86/def-TZVP | -920.900635 | 0.126969 | -920.773666 | 234.9 | 0.051317 | -920.849318 | 251.9 | 0 |
| | BP86/def2-TZVPP | -920.940491 | 0.126797 | -920.813694 | 260.0 | 0.051106 | -920.889385 | 253.0 | 0 |

| | | | | | | | | | |
|---|---|---|---|---|---|---|---|---|---|
| | BP86/def2-TZVPPD | -920.943225 | 0.125669 | -920.817556 | 259.7 | 0.052820 | -920.890405 | 252.6 | 1 |
| | B3LYP/def2-TZVPPD | -920.395797 | 0.129116 | -920.266681 | 258.3 | 0.057846 | -920.337951 | 251.1 | 1 |
| | B3LYP/6-311+G(d,p) | | | -920.655992 | 256.5 | | -920.729905 | 249.3 | 0 |
| | G4(MP2) | | | -919.392932 | 256.9 | | -919.467218 | 249.8 | 0 |
| T2; protonated on CN-1; syn-syn | BP86/def-TZVP | -920.899040 | 0.105182 | -920.793859 | 271.2 | 0.051588 | -920.847452 | 250.6 | 0 |
| T2; protonated on CN-1; rather anti-syn | BP86/def-TZVP | -920.898872 | 0.105224 | -920.793649 | 271.5 | 0.051486 | -920.847386 | 250.7 | 0 |
| T2; protonated on CN-2; syn-syn | BP86/def-TZVP | -920.899030 | 0.105162 | -920.793868 | 271.2 | 0.051531 | -920.847499 | 250.6 | 0 |
| T2; protonated on CN-2; anti-syn | BP86/def-TZVP | -920.900642 | 0.105228 | -920.795414 | 272.6 | 0.051384 | -920.849258 | 251.8 | 0 |
| T2; protonated on CN-2; syn-anti | BP86/def-TZVP | -920.898869 | 0.105221 | -920.793648 | 271.5 | 0.051485 | -920.847384 | 250.7 | 0 |
| T2; protonated on CN-2; anti-anti | BP86/def-TZVP | -920.898867 | 0.105212 | -920.793655 | 272.6 | 0.051367 | -920.847500 | 251.5 | 0 |
| T4; cis-anti | BP86/def-TZVP | -920.868716 | 0.126334 | -920.742382 | 239.3 | 0.049523 | -920.819194 | 233.0 | 0 |
| T4; cis-syn | BP86/def-TZVP | -920.870921 | 0.126332 | -920.744589 | 240.3 | 0.050104 | -920.820817 | 233.9 | 0 |
| T4; trans-anti | BP86/def-TZVP | -920.867521 | 0.126327 | -920.741194 | 239.6 | 0.049105 | -920.818416 | 233.2 | 0 |
| T4; trans-syn | BP86/def-TZVP | -920.871338 | 0.126363 | -920.744975 | 240.9 | 0.050045 | -920.821293 | 234.3 | 0 |
| T5; cis-anti | BP86/def-TZVP | -920.879248 | 0.126479 | -920.752769 | 245.8 | 0.050980 | -920.828268 | 238.7 | 0 |
| T5; cis-anti | BP86/def-TZVP | -920.879250 | 0.126483 | -920.752767 | 245.8 | 0.051020 | -920.828230 | 238.6 | 0 |
| T5; cis-syn | BP86/def-TZVP | -920.882403 | 0.126559 | -920.755844 | 247.3 | 0.051861 | -920.830542 | 240.1 | 0 |
| T5; trans-syn | BP86/def-TZVP | -920.879261 | 0.126487 | -920.752774 | 245.8 | 0.050956 | -920.828305 | 238.7 | 0 |
| T5; trans-anti | BP86/def-TZVP | -920.882410 | 0.126563 | -920.755847 | 248.8 | 0.051841 | -920.830569 | 240.8 | 0 |
| T6; anti-syn | BP86/def-TZVP | -920.878374 | 0.126397 | -920.751977 | 245.3 | 0.050228 | -920.828146 | 238.6 | 0 |
| T6; syn-syn | BP86/def-TZVP | -920.884890 | 0.126515 | -920.758375 | 248.9 | 0.051292 | -920.833598 | 241.9 | 0 |
| T6; anti-anti | BP86/def-TZVP | -920.881512 | 0.126457 | -920.755055 | 248.3 | 0.051020 | -920.830492 | 240.8 | 0 |
| T6; syn-anti | BP86/def-TZVP | -920.881525 | 0.126455 | -920.755070 | 247.3 | 0.048885 | -920.832640 | 241.4 | 0 |
| **13** | | | | | | | | | |
| Anion | | | | | | | | | |
| anti-syn-anti | BP86/def-TZVP | -1182.441154 | 0.150607 | -1182.290547 | | 0.062313 | -1182.378841 | | 0 |
| anti-syn-syn | BP86/def-TZVP | -1182.439360 | 0.150622 | -1182.288738 | | 0.061437 | -1182.377923 | | 0 |
| syn-syn-syn | BP86/def-TZVP | -1182.438355 | 0.150622 | -1182.287733 | | 0.062441 | -1182.375914 | | 0 |
| anti-anti-anti | BP86/def-TZVP | -1182.441940 | 0.150625 | -1182.291315 | | 0.061703 | -1182.380236 | | 0 |
| | BP86/def2-TZVPP | -1182.491515 | 0.150464 | -1182.341051 | | 0.061578 | -1182.429937 | | 0 |
| | BP86/def2-TZVPPD | -1182.495503 | 0.146694 | -1182.348809 | | 0.064213 | -1182.431290 | | 3 |
| | B3LYP/def2-TZVPPD | -1181.789127 | 0.152000 | -1181.637127 | | 0.069697 | -1181.719430 | | 2 |
| | B3LYP/6-311+G(d,p) | | | -1182.138973 | | | -1182.225550 | | 0 |
| | G4(MP2) | | | -1180.522159 | | | -1180.60907 | | 0 |
| Neutral | | | | | | | | | |
| T2, anti-anti-syn | BP86/def-TZVP | -1182.856183 | 0.161547 | -1182.694636 | 255.0 | 0.072174 | -1182.784009 | 248.0 | 0 |
| T2, anti-anti-anti | BP86/def-TZVP | -1182.858143 | 0.161563 | -1182.696580 | 255.8 | 0.071907 | -1182.786235 | 248.5 | 0 |
| | BP86/def2-TZVPP | -1182.909556 | 0.161371 | -1182.748185 | 257.0 | 0.071652 | -1182.837904 | 249.7 | 0 |

| | | | | | | | | | |
|---|---|---|---|---|---|---|---|---|---|
| | BP86/def2-TZVPPD | -1182.913153 | 0.157753 | -1182.755400 | 256.6 | 0.074609 | -1182.838544 | 249.3 | 3 |
| | B3LYP/def2-TZVPPD | -1182.204831 | 0.163367 | -1182.041464 | 255.2 | 0.079639 | -1182.125192 | 248.3 | 2 |
| | B3LYP/6-311+G(d,p) | | | -1182.540316 | 253.3 | | -1182.627589 | 246.0 | 0 |
| | G4(MP2) | | | -1180.925138 | 254.4 | | -1181.012757 | 247.0 | 0 |
| T2, anti-syn-syn | BP86/def-TZVP | -1182.853481 | 0.133972 | -1182.719509 | 271.8 | 0.071292 | -1182.782189 | 247.4 | 0 |
| T2, syn-syn-syn | BP86/def-TZVP | -1182.851293 | 0.133979 | -1182.717314 | 271.0 | 0.070890 | -1182.780403 | 247.5 | 0 |
| T4, trans-syn-anti | BP86/def-TZVP | -1182.828096 | 0.161027 | -1182.667069 | 238.9 | 0.071031 | -1182.757065 | 231.6 | 0 |
| T4; cis-syn-anti | BP86/def-TZVP | -1182.826657 | 0.160997 | -1182.665660 | 238.0 | 0.070428 | -1182.756229 | 231.1 | 0 |
| T4; trans-syn-syn | BP86/def-TZVP | -1182.825689 | 0.160988 | -1182.664701 | 237.4 | 0.069339 | -1182.756350 | 231.2 | 0 |
| T4; cis-syn-syn (symmetrical) | BP86/def-TZVP | -1182.824790 | 0.161013 | -1182.663777 | 237.4 | 0.071216 | -1182.753574 | 230.7 | 0 |
| T4; trans-anti-anti (symmetrical) | BP86/def-TZVP | -1182.827499 | 0.160992 | -1182.666507 | 236.9 | 0.070393 | -1182.757106 | 230.2 | 0 |
| T4; cis-anti-anti | BP86/def-TZVP | -1182.827008 | 0.160928 | -1182.666080 | 237.1 | 0.070756 | -1182.756252 | 230.5 | 0 |
| T4; trans-anti-syn | BP86/def-TZVP | -1182.824841 | 0.160906 | -1182.663935 | 235.8 | 0.070358 | -1182.754483 | 229.4 | 0 |
| T4; cis-anti-syn | BP86/def-TZVP | -1182.823087 | 0.160909 | -1182.662178 | 235.8 | 0.069331 | -1182.753756 | 229.6 | 0 |
| T5; trans-(anti)-anti | BP86/def-TZVP | -1182.839418 | 0.161154 | -1182.678264 | 244.8 | 0.072996 | -1182.766422 | 236.9 | 0 |
| T5; cis-syn-anti | BP86/def-TZVP | -1182.829673 | 0.160997 | -1182.668676 | 238.3 | 0.072226 | -1182.757447 | 230.4 | 0 |
| T5; trans-(anti)-syn | BP86/def-TZVP | -1182.836788 | 0.161077 | -1182.675711 | 244.9 | 0.072117 | -1182.764671 | 237.7 | 0 |
| T5; cis-syn-(syn) | BP86/def-TZVP | -1182.828933 | 0.161002 | -1182.667931 | 239.4 | 0.072043 | -1182.756890 | 231.5 | 0 |
| T5; trans-anti-anti | BP86/def-TZVP | -1182.838821 | 0.161164 | -1182.677657 | 244.4 | 0.072667 | -1182.766154 | 236.8 | 0 |
| T5; cis-anti-anti | BP86/def-TZVP | -1182.830267 | 0.160984 | -1182.669283 | 240.3 | 0.071737 | -1182.758530 | 232.6 | 0 |
| T5; trans-anti-syn | BP86/def-TZVP | -1182.835720 | 0.161049 | -1182.674671 | 242.5 | 0.071688 | -1182.764032 | 235.4 | 0 |
| T5; cis-anti-(anti) | BP86/def-TZVP | -1182.830196 | 0.161009 | -1182.669187 | 240.2 | 0.072142 | -1182.758054 | 232.3 | 0 |
| T6; trans-syn-anti | BP86/def-TZVP | -1182.837019 | 0.161070 | -1182.675949 | 243.3 | 0.071753 | -1182.765266 | 236.2 | 0 |
| T6; cis-(anti)-anti | BP86/def-TZVP | -1182.838672 | 0.161136 | -1182.677536 | 245.5 | 0.072262 | -1182.766410 | 237.5 | 0 |
| T6; trans-syn-(syn) | BP86/def-TZVP | -1182.836061 | 0.161039 | -1182.675022 | 243.9 | 0.071390 | -1182.764671 | 236.4 | 0 |
| T6; cis-syn-syn (symmetrical) | BP86/def-TZVP | -1182.835070 | 0.161092 | -1182.673978 | 243.9 | 0.071627 | -1182.763443 | 236.9 | 0 |
| T6; trans-anti-anti (symmetrical) | BP86/def-TZVP | -1182.835177 | 0.161021 | -1182.674156 | 241.7 | 0.069689 | -1182.765488 | 235.5 | 0 |
| T6; cis-anti-anti | BP86/def-TZVP | -1182.840422 | 0.161110 | -1182.679312 | 245.4 | 0.072262 | -1182.768160 | 238.0 | 0 |
| T6; trans-anti-(anti) | BP86/def-TZVP | -1182.834598 | 0.160997 | -1182.673601 | 241.4 | 0.069680 | -1182.764918 | 235.1 | 0 |
| T6; cis-ani-syn | BP86/def-TZVP | -1182.835817 | 0.161050 | -1182.674767 | 243.7 | 0.070796 | -1182.765021 | 236.6 | 0 |
| | | | | | | | | | |
| **14** | | | | | | | | | |
| Anion | | | | | | | | | |
| conformer I | BP86/def-TZVP | -1444.394467 | 0.185116 | -1444.209351 | | 0.085235 | -1444.309232 | | 0 |
| conformer II | BP86/def-TZVP | -1444.394606 | 0.185057 | -1444.209549 | | 0.084463 | -1444.310143 | | 0 |
| | BP86/def2-TZVPP | -1444.455659 | 0.184848 | -1444.270811 | | 0.084199 | -1444.371460 | | 0 |
| conformer III; most stable from CosmoConf | BP86/def-TZVP | -1444.398421 | 0.185087 | -1444.213334 | | 0.085113 | -1444.313308 | | 0 |
| | BP86/def2-TZVPP | -1444.459443 | 0.184893 | -1444.274550 | | 0.084896 | -1444.374547 | | 0 |
| | BP86/def2-TZVPPD | -1444.464317 | 0.172971 | -1444.291346 | | 0.082882 | -1444.381435 | | 8 |

| | | | | | | | | | |
|---|---|---|---|---|---|---|---|---|---|
| | B3LYP/6-311+G(d,p) | | | -1444.022448 | | | -1444.119902 | | 0 |
| conformer IV | BP86/def-TZVP | -1444.398374 | 0.185060 | -1444.213314 | | 0.085023 | -1444.313351 | | 0 |
| Neutral | | | | | | | | | |
| T4; conformer I | BP86/def-TZVP | -1444.773503 | 0.195401 | -1444.578102 | 232.9 | 0.092612 | -1444.680890 | 226.9 | 0 |
| T4; conformer I | BP86/def-TZVP | -1444.777754 | 0.195439 | -1444.582315 | 235.5 | 0.093606 | -1444.684148 | 229.0 | 0 |
| T4; conformer I | BP86/def-TZVP | -1444.777739 | 0.195433 | -1444.582306 | 235.5 | 0.093544 | -1444.684195 | 229.0 | 0 |
| T4; conformer I | BP86/def-TZVP | -1444.777746 | 0.195435 | -1444.582311 | 235.5 | 0.093557 | -1444.684189 | 229.0 | 0 |
| T4; conformer I | BP86/def-TZVP | -1444.777751 | 0.195439 | -1444.582312 | 235.5 | 0.093626 | -1444.684125 | 229.0 | 0 |
| T4; conformer II | BP86/def-TZVP | -1444.776399 | 0.195410 | -1444.580989 | 234.6 | 0.092180 | -1444.684219 | 228.5 | 0 |
| T4; conformer II | BP86/def-TZVP | -1444.776462 | 0.195377 | -1444.581085 | 234.6 | 0.092043 | -1444.684419 | 228.6 | 0 |
| T4; conformer II | BP86/def-TZVP | -1444.778075 | 0.195435 | -1444.582640 | 235.6 | 0.093099 | -1444.684976 | 228.9 | 0 |
| T4; conformer II | BP86/def-TZVP | -1444.774364 | 0.195388 | -1444.578976 | 233.3 | 0.092250 | -1444.682114 | 227.1 | 0 |
| T4; conformer III | BP86/def-TZVP | -1444.780037 | 0.195406 | -1444.584631 | 234.5 | 0.092816 | -1444.687221 | 228.4 | 0 |
| T4; conformer III | BP86/def-TZVP | -1444.780061 | 0.195416 | -1444.584645 | 234.5 | 0.092839 | -1444.687222 | 228.4 | 0 |
| T4; conformer III | BP86/def-TZVP | -1444.780031 | 0.195411 | -1444.584620 | 234.5 | 0.092746 | -1444.687285 | 228.4 | 0 |
| T4; conformer III | BP86/def-TZVP | -1444.780061 | 0.195425 | -1444.584636 | 234.5 | 0.092879 | -1444.687182 | 228.3 | 0 |
| T4; conformer IV | BP86/def-TZVP | -1444.780005 | 0.195393 | -1444.584612 | 234.5 | 0.092752 | -1444.687253 | 228.3 | 0 |
| T4; conformer IV | BP86/def-TZVP | -1444.780019 | 0.195394 | -1444.584625 | 234.5 | 0.092813 | -1444.687206 | 228.3 | 0 |
| T4; conformer IV | BP86/def-TZVP | -1444.780011 | 0.195390 | -1444.584621 | 234.5 | 0.092676 | -1444.687335 | 228.4 | 0 |
| T4; conformer IV | BP86/def-TZVP | -1444.780030 | 0.195399 | -1444.584631 | 234.5 | 0.092932 | -1444.687098 | 228.3 | 0 |
| T5; conformer I | BP86/def-TZVP | -1444.782104 | 0.195512 | -1444.586592 | 238.2 | 0.094211 | -1444.687893 | 231.3 | 0 |
| T5; conformer I | BP86/def-TZVP | -1444.781788 | 0.195468 | -1444.586320 | 238.0 | 0.093430 | -1444.688358 | 231.6 | 0 |
| T5; conformer I | BP86/def-TZVP | -1444.782106 | 0.195512 | -1444.586594 | 238.2 | 0.094322 | -1444.687784 | 231.3 | 0 |
| T5; conformer I | BP86/def-TZVP | -1444.782121 | 0.195520 | -1444.586601 | 238.2 | 0.094326 | -1444.687795 | 231.3 | 0 |
| T5; conformer II | BP86/def-TZVP | -1444.779463 | 0.195391 | -1444.584072 | 236.5 | 0.093368 | -1444.686095 | 229.6 | 0 |
| T5; conformer II | BP86/def-TZVP | -1444.777941 | 0.195409 | -1444.582532 | 235.5 | 0.092541 | -1444.685400 | 229.2 | 0 |
| T5; conformer II | BP86/def-TZVP | -1444.776606 | 0.195335 | -1444.581271 | 234.7 | 0.092698 | -1444.683908 | 228.3 | 0 |
| T5; conformer II | BP86/def-TZVP | -1444.777906 | 0.195387 | -1444.582519 | 235.5 | 0.093810 | -1444.684096 | 228.4 | 0 |
| T5; conformer III | BP86/def-TZVP | -1444.782043 | 0.195481 | -1444.586562 | 235.7 | 0.093777 | -1444.688266 | 229.0 | 0 |
| T5; conformer III | BP86/def-TZVP | -1444.782010 | 0.195459 | -1444.586551 | 235.7 | 0.093662 | -1444.688348 | 229.1 | 0 |
| T5; conformer III | BP86/def-TZVP | -1444.782029 | 0.195452 | -1444.586577 | 235.7 | 0.093640 | -1444.688389 | 229.1 | 0 |
| T5; conformer III | BP86/def-TZVP | -1444.782012 | 0.195470 | -1444.586542 | 235.7 | 0.093732 | -1444.688280 | 229.0 | 0 |
| T5; conformer IV | BP86/def-TZVP | -1444.782005 | 0.195466 | -1444.586539 | 235.7 | 0.093711 | -1444.688294 | 229.0 | 0 |
| T5; conformer IV | BP86/def-TZVP | -1444.781999 | 0.195457 | -1444.586542 | 235.7 | 0.093727 | -1444.688272 | 229.0 | 0 |
| T5; conformer IV | BP86/def-TZVP | -1444.782009 | 0.195445 | -1444.586564 | 235.7 | 0.093702 | -1444.688307 | 229.0 | 0 |
| T5; conformer IV | BP86/def-TZVP | -1444.782003 | 0.195454 | -1444.586549 | 235.7 | 0.093619 | -1444.688384 | 229.1 | 0 |
| T6; conformer I | BP86/def-TZVP | -1444.784589 | 0.195525 | -1444.589064 | 239.8 | 0.093804 | -1444.690785 | 233.1 | 0 |
| T6; conformer I | BP86/def-TZVP | -1444.784442 | 0.195508 | -1444.588934 | 239.7 | 0.093707 | -1444.690735 | 233.1 | 0 |
| T6; conformer I | BP86/def-TZVP | -1444.784664 | 0.195528 | -1444.589136 | 239.8 | 0.093856 | -1444.690808 | 233.2 | 0 |

| | | | | | | | | | |
|---|---|---|---|---|---|---|---|---|---|
| T6; conformer I | BP86/def-TZVP | -1444.784659 | 0.195524 | -1444.589135 | 239.8 | 0.093813 | -1444.690846 | 233.2 | 0 |
| T6; conformer II | BP86/def-TZVP | -1444.786009 | 0.195494 | -1444.590515 | 240.5 | 0.093413 | -1444.692596 | 233.7 | 0 |
| T6; conformer II | BP86/def-TZVP | -1444.787859 | 0.195532 | -1444.592327 | 241.7 | 0.093479 | -1444.694380 | 234.8 | 0 |
| T6; conformer II | BP86/def2-TZVPP | -1444.851112 | 0.195292 | -1444.655820 | 243.1 | 0.093143 | -1444.757969 | 236.3 | 0 |
| T6; conformer II | BP86/def-TZVP | -1444.785172 | 0.195502 | -1444.589670 | 240.0 | 0.093229 | -1444.691943 | 233.3 | 0 |
| T6; conformer II | BP86/def-TZVP | -1444.782258 | 0.195434 | -1444.586824 | 238.2 | 0.092437 | -1444.689821 | 232.0 | 0 |
| T6; conformer III | BP86/def-TZVP | -1444.788364 | 0.195505 | -1444.592859 | 239.6 | 0.093675 | -1444.694689 | 233.0 | 0 |
| T6; conformer III | BP86/def-TZVP | -1444.788330 | 0.195495 | -1444.592835 | 239.6 | 0.093627 | -1444.694703 | 233.1 | 0 |
| T6; conformer III | BP86/def-TZVP | -1444.788342 | 0.195504 | -1444.592838 | 239.6 | 0.093634 | -1444.694708 | 233.1 | 0 |
| T6; conformer III | BP86/def2-TZVPP | -1444.851336 | 0.195272 | -1444.656064 | 240.9 | 0.093222 | -1444.758114 | 234.4 | 0 |
| T6; conformer III | BP86/def2-TZVPPD | -1444.855872 | 0.181074 | -1444.674798 | 242.1 | 0.094179 | -1444.761693 | 232.3 | 10 |
| T6; conformer III | B3LYP/6-311+G(d,p) | | | -1444.396548 | 236.2 | | -1444.495392 | 229.3 | 0 |
| T6; conformer III | BP86/def-TZVP | -1444.788306 | 0.195499 | -1444.592807 | 239.6 | 0.093513 | -1444.694793 | 233.1 | 0 |
| T6; conformer IV | BP86/def-TZVP | -1444.788302 | 0.195472 | -1444.592830 | 239.6 | 0.093564 | -1444.694738 | 233.0 | 0 |
| T6; conformer IV | BP86/def-TZVP | -1444.788326 | 0.195490 | -1444.592836 | 239.6 | 0.093656 | -1444.694670 | 233.0 | 0 |
| T6; conformer IV | BP86/def-TZVP | -1444.788334 | 0.195487 | -1444.592847 | 239.6 | 0.093615 | -1444.694719 | 233.0 | 0 |
| T6; conformer IV | BP86/def-TZVP | -1444.788288 | 0.195485 | -1444.592803 | 239.6 | 0.093496 | -1444.694792 | 233.1 | 0 |
| **15** | | | | | | | | | |
| Anion | | | | | | | | | |
| | BP86/def-TZVP | -92.891539 | 0.007963 | -92.883576 | | -0.0143861 | -92.905925 | | 0 |
| | B3LYP/def2-TZVPPD | -92.894504 | 0.007962 | -92.886542 | | -0.014426 | -92.908930 | | 0 |
| | B3LYP/6-311+G(d,p) | | | -92.880338 | | | -92.902682 | | 0 |
| Neutral | | | | | | | | | |
| C-N-H | BP86/def-TZVP | -93.439333 | 0.018078 | -93.421255 | 338.87468 | 0.0009865 | -93.438347 | 327.8 | 0 |
| H-C-N | BP86/def-TZVP | -93.463095 | 0.017952 | -93.445143 | 353.86422 | 0.0022777 | -93.460817 | 341.9 | 0 |
| | B3LYP/def2-TZVPPD | -93.466651 | 0.020417 | -93.446234 | 352.68775 | 0.005538 | -93.461113 | 340.2 | 0 |
| | B3LYP/6-311+G(d,p) | | | -93.434649 | 349.31115 | | -93.457492 | 341.9 | 0 |
| **16** | | | | | | | | | |
| Anion | | | | | | | | | |
| | BP86/def-TZVP | -354.899269 | 0.041981 | -354.857288 | | 0.001916 | -354.897353 | | 0 |
| | B3LYP/def2-TZVPPD | -354.914437 | 0.041953 | -354.872484 | | 0.001571 | -354.912866 | | 0 |
| | B3LYP/6-311+G(d,p) | | | -354.811547 | | | -354.851410 | | 0 |
| Neutal | | | | | | | | | |
| T1; $C(CN)_2$=CH(CN) | BP86/def-TZVP | -355.437345 | 0.054687 | -355.382658 | 331.15071 | 0.014283 | -355.423061 | 323.6 | 0 |
| | B3LYP/def2-TZVPPD | -355.452729 | 0.054632 | -355.398097 | 331.30291 | 0.013783 | -355.438946 | 323.8 | 0 |
| | B3LYP/6-311+G(d,p) | | | -355.336145 | 330.66624 | | -355.376488 | 323.2 | 0 |
| T4; $C(CN\text{-}H)_2$=C(CN) | BP86/def-TZVP | -355.353640 | 0.052143 | -355.301497 | 280.22258 | 0.010405 | -355.343235 | 273.5 | 0 |

| | | | | | | | | | |
|---|---|---|---|---|---|---|---|---|---|
| T6; $C(CN)_2{=}C(CN\text{-}H)$ | BP86/def-TZVP | -355.393389 | 0.053931 | -355.339458 | 304.04308 | 0.012341 | -355.381049 | 297.2 | 0 |
| T5; $C(CN\text{-}H)_2{=}C(CN)$ | BP86/def-TZVP | -355.348482 | 0.052442 | -355.296040 | 276.79807 | 0.009942 | -355.338540 | 270.6 | 0 |
| | | | | | | | | | |
| **17** | | | | | | | | | |
| Dianion | | | | | | | | | |
| $[B_{12}(CN)_{12}]^{2-}$ | BP86/def-TZVP | -1413.480680 | 0.188055 | -1413.292625 | | 0.091658 | -1413.389022 | | 3 |
| | BP86/def-TZVP | -1413.480695 | 0.188084 | -1413.292611 | | 0.091703 | -1413.388992 | | 3 |
| | B3LYP/def2-TZVPPD | -1413.536355 | 0.186733 | -1413.349622 | | 0.089620 | -1413.446735 | | 3 |
| | B3LYP/6-311+G(d,p) | | | -1413.152635 | | | -1413.248443 | | |
| Anion | | | | | | | | | |
| $[B_{12}(CN)_{12}]^{-}$, protonated on CN | BP86/def-TZVP | -1413.964512 | 0.203011 | -1413.761501 | 295.7 | 0.100872 | -1413.863640 | 291.5 | 0 |
| | B3LYP/def2-TZVPPD | -1414.021731 | 0.198238 | -1413.823493 | 298.8 | 0.099193 | -1413.922538 | 292.3 | 3 |
| | B3LYP/6-311+G(d,p) | | | -1413.620604 | 295.1 | | -1413.718562 | 288.7 | 0 |
| | | | | | | | | | |
| **18** | | | | | | | | | |
| Neutral | | | | | | | | | |
| $B_{12}(CN)_{12}H_2$, H-s dide by side | BP86/def-TZVP | -1414.350481 | 0.212976 | -1414.137505 | 237.4 | 0.108904 | -1414.241577 | 230.9 | 0 |
| $B_{12}(CN)_{12}H_2$, 1 B between two H-s | BP86/def-TZVP | -1414.362171 | 0.210972 | -1414.151199 | 246.0 | 0.109079 | -1414.253092 | 238.1 | 2 |
| | BP86/def-TZVP | -1414.362171 | 0.210013 | -1414.152158 | | | -1414.362171 | | 3 |
| $B_{12}(CN)_{12}H_2$, 2 B-s between two H-s | BP86/def-TZVP | -1414.365218 | 0.213296 | -1414.151922 | 246.5 | 0.109359 | -1414.255859 | 239.8 | 0 |
| | B3LYP/def2-TZVPPD | -1414.423871 | 0.210204 | -1414.213667 | 246.3 | 0.106791 | -1414.317080 | 241.3 | 2 |
| | B3LYP/6-311+G(d,p) | | | -1414.006915 | 243.9 | | -1414.106517 | 237.2 | |
| | | | | | | | | | |
| **19** | | | | | | | | | |
| Anion | | | | | | | | | |
| $[CB_{11}(CN)_{12}]^{-}$ | BP86/def-TZVP | -1426.559923 | 0.192538 | -1426.367385 | | 0.093785 | -1426.466138 | | 0 |
| | B3LYP/def2-TZVPPD | -1426.616784 | 0.191696 | -1426.425088 | | 0.090700 | -1426.526084 | | 0 |
| | B3LYP/6-311+G(d,p) | | | -1426.217878 | | | -1426.314578 | | 0 |
| Neutral | | | | | | | | | |
| $CB_{11}(CN)_{12}H$, H on CN that is on C | BP86/def-TZVP | -1426.917444 | 0.202578 | -1426.714866 | 219.5 | 0.101714 | -1426.815730 | 213.1 | 0 |
| $CB_{11}(CN)_{12}H$, H on CN that is on 1st row B | BP86/def-TZVP | -1426.930505 | 0.203348 | -1426.727157 | 227.2 | 0.103048 | -1426.827457 | 220.5 | 0 |
| $CB_{11}(CN)_{12}H$, H on CN that is on 2nd row B | BP86/def-TZVP | -1426.938914 | 0.203342 | -1426.735572 | 232.5 | 0.102888 | -1426.836026 | 225.8 | 0 |
| $CB_{11}(CN)_{12}H$, H on CN that is on opposite C | BP86/def-TZVP | -1426.941727 | 0.203149 | -1426.738578 | 234.4 | 0.102169 | -1426.839558 | 228.0 | 0 |
| | B3LYP/def2-TZVPPD | -1427.000134 | 0.202346 | -1426.797788 | 235.4 | 0.098932 | -1426.901202 | 229.1 | 0 |
| | B3LYP/6-311+G(d,p) | | | -1426.584522 | 231.6 | | -1426.683007 | 224.9 | 0 |

**Table S10**. DataDOI data repository filenames of title compounds in referred folders. See http://dx.doi.org/10.23673/re-436.

| Compound | Basis set | GEO file | FR file | Folder | Cosmo file in CN_Cosmo folder |
|---|---|---|---|---|---|
| **1** | | | | | |
| Anion | | | | | |
| | BP86/def-TZVP | job_GEO_238 | job_SP_239 | CN_2 | |
| | BP86/def2-TZVPP | job_GEO_491 | job_SP_497 | CN_1 | COSMO-BP-TZVPD-FINE_17 |
| | BP86/def2-TZVPPD | job_GEO_715 | job_SP_717 | CN_1 | |
| | B3LYP/def2-TZVPPD | job_GEO_719 | job_SP_723 | CN_1 | |
| | B3LYP/6-311+G(d,p) | JOB_1000 | | CN_GAU | |
| | G4(MP2) | JOB_1000_1 | | CN_GAU | |
| | W1RO | JOB_1000_2 | | CN_GAU | |
| | CBS-APNO | JOB_1000_3 | | CN_GAU | |
| Neutral | | | | | |
| T1 | BP86/def-TZVP | job_GEO_242 | job_SP_243 | CN_2 | |
| T2 | BP86/def-TZVP | job_GEO_361 | job_SP_362 | CN_2 | |
| | BP86/def2-TZVPP | job_GEO_492 | job_SP_498 | CN_1 | COSMO-BP-TZVPD-FINE_19 |
| | BP86/def2-TZVPPD | job_GEO_716 | job_SP_718 | CN_1 | |
| | B3LYP/def2-TZVPPD | job_GEO_720 | job_SP_724 | CN_1 | |
| | B3LYP/6-311+G(d,p) | JOB_1001 | | CN_GAU | |
| | G4(MP2) | JOB_1001_1 | | CN_GAU | |
| | W1RO | JOB_1001_2 | | CN_GAU | |
| | CBS-APNO | JOB_1001_3 | | CN_GAU | |
| **2** | | | | | |
| Anion | | | | | |
| | BP86/def-TZVP | job_GEO_223 | job_SP_231 | CN_2 | |
| | BP86/def2-TZVPP | job_GEO_501 | job_SP_503 | CN_1 | COSMO-BP-TZVPD-FINE_21 |
| | BP86/def2-TZVPPD | job_GEO_725 | job_SP_729 | CN_1 | |
| | B3LYP/def2-TZVPPD | job_GEO_727 | job_SP_731 | CN_1 | |
| | B3LYP/6-311+G(d,p) | JOB_1002 | | CN_GAU | |
| | G4(MP2) | JOB_1002_1 | | CN_GAU | |
| | W1RO | JOB_1002_2 | | CN_GAU | |
| | CBS-APNO | JOB_1002_3 | | CN_GAU | |
| Neutral | | | | | |
| T1 | BP86/def-TZVP | job_GEO_224 | job_SP_232 | CN_2 | |
| | BP86/def2-TZVPP | job_GEO_502 | job_SP_505 | CN_1 | COSMO-BP-TZVPD-FINE_23 |
| | BP86/def2-TZVPPD | job_GEO_726 | job_SP_730 | CN_1 | |
| | B3LYP/def2-TZVPPD | job_GEO_728 | job_SP_732 | CN_1 | |

| | | | | | |
|---|---|---|---|---|---|
| | B3LYP/6-311+G(d,p) | JOB_1003 | | CN_GAU | |
| | G4(MP2) | JOB_1003_1 | | CN_GAU | |
| | W1RO | JOB_1003_2 | | CN_GAU | |
| | CBS-APNO | JOB_1003_3 | | CN_GAU | |
| T3; eclipsed(H) | BP86/def-TZVP | job_GEO_225 | job_SP_233 | CN_2 | |
| T3; syn(H) | BP86/def-TZVP | job_GEO_78 | job_SP_80 | CN_1 | |
| T3; anti(H) | BP86/def-TZVP | job_GEO_82 | job_SP_83 | CN_1 | |
| T4 | BP86/def-TZVP | job_GEO_32 | job_SP_34 | CN_1 | COSMO-BP-TZVPD-FINE_316 |
| T5 | BP86/def-TZVP | job_GEO_226 | job_SP_234 | CN_2 | COSMO-BP-TZVPD-FINE_314 |
| T6 | BP86/def-TZVP | job_GEO_33 | job_SP_35 | CN_1 | |
| | | | | | |
| **3** | | | | | |
| Anion | | | | | |
| | BP86/def-TZVP | job_GEO_363 | job_SP_364 | CN_2 | |
| | BP86/def2-TZVPP | job_GEO_506 | job_SP_508 | CN_1 | COSMO-BP-TZVPD-FINE_26 |
| | BP86/def2-TZVPPD | job_GEO_733 | job_SP_760 | CN_1 | |
| | B3LYP/def2-TZVPPD | job_GEO_734 | job_SP_761 | CN_1 | |
| | B3LYP/6-311+G(d,p) | JOB_1004 | | CN_GAU | |
| | G4(MP2) | JOB_1004_1 | | CN_GAU | |
| | W1RO | JOB_1004_2 | | CN_GAU | |
| | CBS-APNO | JOB_1004_3 | | CN_GAU | |
| Neutral | | | | | |
| T1 | BP86/def-TZVP | job_GEO_247 | job_SP_250 | CN_2 | |
| T2 | BP86/def-TZVP | job_GEO_248 | job_SP_251 | CN_2 | |
| | BP86/def2-TZVPP | job_GEO_507 | job_SP_509 | CN_1 | COSMO-BP-TZVPD-FINE_28 |
| | BP86/def2-TZVPPD | job_GEO_735 | job_SP_762 | CN_1 | |
| | B3LYP/def2-TZVPPD | job_GEO_736 | job_SP_763 | CN_1 | |
| | B3LYP/6-311+G(d,p) | JOB_1005 | | CN_GAU | |
| | G4(MP2) | JOB_1005_1 | | CN_GAU | |
| | W1RO | JOB_1005_2 | | CN_GAU | |
| | CBS-APNO | JOB_1005_3 | | CN_GAU | |
| | | | | | |
| **4** | | | | | |
| Anion | | | | | |
| anti | BP86/def-TZVP | job_GEO_254 | job_SP_263 | CN_2 | |
| | BP86/def2-TZVPP | job_GEO_510 | job_SP_515 | CN_1 | COSMO-BP-TZVPD-FINE_116 |
| | BP86/def2-TZVPPD | job_GEO_737 | job_SP_776 | CN_1 | |
| | B3LYP/def2-TZVPPD | job_GEO_738 | job_SP_777 | CN_1 | |
| | B3LYP/6-311+G(d,p) | JOB_1006 | | CN_GAU | |

| | | | | | |
|---|---|---|---|---|---|
| | G4(MP2) | JOB_1006_1 | | CN_GAU | |
| | W1RO | JOB_1006_2 | | CN_GAU | |
| | CBS-APNO | JOB_1006_3 | | CN_GAU | |
| syn | BP86/def-TZVP | job_GEO_332 | job_SP_340 | CN_2 | |
| | BP86/def2-TZVPP | job_GEO_511 | job_SP_516 | CN_1 | COSMO-BP-TZVPD-FINE_30 |
| Neutral | | | | | |
| T1; syn | BP86/def-TZVP | job_GEO_255 | job_SP_264 | CN_2 | |
| T1; anti | BP86/def-TZVP | job_GEO_333 | job_SP_341 | CN_2 | |
| T2; anti | BP86/def-TZVP | job_GEO_258 | job_SP_267 | CN_2 | |
| | BP86/def2-TZVPP | job_GEO_514 | job_SP_518 | CN_1 | COSMO-BP-TZVPD-FINE_118 |
| | BP86/def2-TZVPPD | job_GEO_739 | job_SP_778 | CN_1 | |
| | B3LYP/def2-TZVPPD | job_GEO_740 | job_SP_779 | CN_1 | |
| | B3LYP/6-311+G(d,p) | JOB_1007 | | CN_GAU | |
| | G4(MP2) | JOB_1007_1 | | CN_GAU | |
| | W1RO | JOB_1007_2 | | CN_GAU | |
| | CBS-APNO | JOB_1007_3 | | CN_GAU | |
| T2; syn | BP86/def-TZVP | job_GEO_335 | job_SP_343 | CN_2 | |
| | BP86/def2-TZVPP | job_GEO_513 | job_SP_517 | CN_1 | COSMO-BP-TZVPD-FINE_32 |
| T3; trans, eclipsed(H) | BP86/def-TZVP | job_GEO_256 | job_SP_265 | CN_2 | |
| T3; trans, syn(H) | BP86/def-TZVP | job_GEO_85 | job_SP_95 | CN_1 | |
| T3; cis, anti(H) | BP86/def-TZVP | job_GEO_22 | job_SP_23 | CN_1 | |
| T3; cis, syn(H) | BP86/def-TZVP | job_GEO_84 | job_SP_94 | CN_1 | |
| T4; trans | BP86/def-TZVP | job_GEO_257 | job_SP_266 | CN_2 | |
| T4; cis | BP86/def-TZVP | job_GEO_334 | job_SP_342 | CN_2 | |
| T5; trans | BP86/def-TZVP | job_GEO_37 | job_SP_45 | CN_1 | |
| T5; cis | BP86/def-TZVP | job_GEO_38 | job_SP_42 | CN_1 | |
| T6; anti | BP86/def-TZVP | job_GEO_40 | job_SP_44 | CN_1 | |
| T6; syn | BP86/def-TZVP | job_GEO_39 | job_SP_43 | CN_1 | |
| | | | | | |
| **5** | | | | | |
| Anion | | | | | |
| Anion; anti-syn (syn facing forward) | BP86/def-TZVP | job_GEO_259 | job_SP_269 | CN_2 | |
| Anion; anti-syn (syn facing backward) | BP86/def-TZVP | job_GEO_471 | job_SP_475 | CN_1 | COSMO-BP-TZVPD-FINE_120 |
| | BP86/def2-TZVPP | job_GEO_522 | job_SP_524 | CN_1 | COSMO-BP-TZVPD-FINE_53 |
| anti-anti | BP86/def-TZVP | job_GEO_19 | job_SP_20 | CN_1 | COSMO-BP-TZVPD-FINE_122 |
| | BP86/def2-TZVPP | job_GEO_741 | job_SP_770 | CN_1 | |
| | BP86/def2-TZVPPD | job_GEO_742 | job_SP_771 | CN_1 | |
| | B3LYP/def2-TZVPPD | job_GEO_775 | job_SP_794 | CN_1 | |
| | B3LYP/6-311+G(d,p) | JOB_1008 | | CN_GAU | |

| | | | | | |
|---|---|---|---|---|---|
| | G4(MP2) | JOB_1008_1 | | CN_GAU | |
| | CBS-APNO | JOB_1008_3 | | CN_GAU | |
| syn-syn | BP86/def-TZVP | job_GEO_25 | job_SP_26 | CN_1 | COSMO-BP-TZVPD-FINE_124 |
| Neutral | | | | | |
| T1; anti-syn (syn facing forward) | BP86/def-TZVP | job_GEO_472 | job_SP_476 | CN_1 | COSMO-BP-TZVPD-FINE_126 |
| T1; anti-syn (syn facing backward) | BP86/def-TZVP | job_GEO_260 | job_SP_270 | CN_2 | |
| | BP86/def2-TZVPP | job_GEO_523 | job_SP_525 | CN_1 | COSMO-BP-TZVPD-FINE_55 |
| T1; syn-syn | BP86/def-TZVP | job_GEO_27 | job_SP_29 | CN_1 | COSMO-BP-TZVPD-FINE_128 |
| T1; anti-anti | BP86/def-TZVP | job_GEO_795 | job_SP_812 | CN_1 | COSMO-BP-TZVPD-FINE_130 |
| | BP86/def2-TZVPP | job_GEO_744 | job_SP_773 | CN_1 | |
| | BP86/def2-TZVPPD | job_GEO_745 | job_SP_774 | CN_1 | |
| | B3LYP/def2-TZVPPD | job_GEO_758 | job_SP_772 | CN_1 | |
| | B3LYP/6-311+G(d,p) | JOB_1009 | | CN_GAU | |
| | G4(MP2) | JOB_1009_1 | | CN_GAU | |
| | CBS-APNO | JOB_1009_3 | | CN_GAU | |
| T3; cis-anti, eclipsed(H) | BP86/def-TZVP | job_GEO_261 | job_SP_271 | CN_2 | |
| T3; cis-anti, syn(H) | BP86/def-TZVP | job_GEO_91 | job_SP_104 | CN_1 | |
| T3; trans-anti, syn(H) | BP86/def-TZVP | job_GEO_338 | job_SP_349 | CN_2 | |
| T3; trans-anti, eclipsed(H) | BP86/def-TZVP | job_GEO_96 | job_SP_105 | CN_1 | |
| T3; cis-syn, eclipsed(H) | BP86/def-TZVP | job_GEO_75 | job_SP_106 | CN_1 | |
| T3; cis-syn, syn(H) | BP86/def-TZVP | job_GEO_92 | job_SP_107 | CN_1 | |
| T3; trans-syn, syn(H) | BP86/def-TZVP | job_GEO_67 | job_SP_76 | CN_1 | |
| T3; trans-syn, eclipsed(H) | BP86/def-TZVP | job_GEO_93 | job_SP_108 | CN_1 | |
| T4; cis-anti | BP86/def-TZVP | job_GEO_262 | job_SP_272 | CN_2 | |
| T4; trans-anti | BP86/def-TZVP | job_GEO_339 | job_SP_347 | CN_2 | |
| T4; cis-syn | BP86/def-TZVP | job_GEO_51 | job_SP_72 | CN_1 | |
| T4; trans-syn | BP86/def-TZVP | job_GEO_47 | job_SP_59 | CN_1 | |
| T5; trans-anti | BP86/def-TZVP | job_GEO_48 | job_SP_64 | CN_1 | |
| T5; cis-syn | BP86/def-TZVP | job_GEO_49 | job_SP_65 | CN_1 | |
| T5; trans-syn | BP86/def-TZVP | job_GEO_50 | job_SP_57 | CN_1 | |
| T5; cis-anti | BP86/def-TZVP | job_GEO_905 | job_SP_906 | CN_1 | |
| T6; anti-anti | BP86/def-TZVP | job_GEO_60 | job_SP_68 | CN_1 | |
| T6; syn-syn | BP86/def-TZVP | job_GEO_61 | job_SP_73 | CN_1 | |
| T6; anti-syn | BP86/def-TZVP | job_GEO_62 | job_SP_70 | CN_1 | |
| T6; syn-anti | BP86/def-TZVP | job_GEO_63 | job_SP_74 | CN_1 | |
| | | | | | |
| **6** | | | | | |
| Anion | | | | | |
| | BP86/def-TZVP | job_GEO_273 | job_SP_290 | CN_2 | |

| | | | | | |
|---|---|---|---|---|---|
| | BP86/def2-TZVPP | job_GEO_538 | job_SP_540 | CN_1 | COSMO-BP-TZVPD-FINE_38 |
| | BP86/def2-TZVPPD | job_GEO_747 | job_SP_799 | CN_1 | |
| | B3LYP/def2-TZVPPD | job_GEO_748 | job_SP_800 | CN_1 | |
| | B3LYP/6-311+G(d,p) | JOB_1010 | | CN_GAU | |
| | G4(MP2) | JOB_1010_1 | | CN_GAU | |
| | W1RO | JOB_1010_2 | | CN_GAU | |
| | CBS-APNO | JOB_1010_3 | | CN_GAU | |
| Neutral | | | | | |
| T1 | BP86/def-TZVP | job_GEO_274 | job_SP_291 | CN_2 | |
| T2 | BP86/def-TZVP | job_GEO_275 | job_SP_292 | CN_2 | |
| | BP86/def2-TZVPP | job_GEO_539 | job_SP_542 | CN_1 | COSMO-BP-TZVPD-FINE_40 |
| | BP86/def2-TZVPPD | job_GEO_749 | job_SP_801 | CN_1 | |
| | B3LYP/def2-TZVPPD | job_GEO_750 | job_SP_802 | CN_1 | |
| | B3LYP/6-311+G(d,p) | JOB_1011 | | CN_GAU | |
| | G4(MP2) | JOB_1011_1 | | CN_GAU | |
| | W1RO | JOB_1011_2 | | CN_GAU | |
| | CBS-APNO | JOB_1011_3 | | CN_GAU | |
| **7** | | | | | |
| Anion | | | | | |
| | BP86/def-TZVP | job_GEO_276 | job_SP_293 | CN_2 | |
| | BP86/def2-TZVPP | job_GEO_544 | job_SP_570 | CN_1 | COSMO-BP-TZVPD-FINE_57 |
| | BP86/def2-TZVPPD | job_GEO_751 | job_SP_803 | CN_1 | |
| | B3LYP/def2-TZVPPD | job_GEO_752 | job_SP_804 | CN_1 | |
| | B3LYP/6-311+G(d,p) | JOB_1012 | | CN_GAU | |
| | G4(MP2) | JOB_1012_1 | | CN_GAU | |
| | W1RO | JOB_1012_2 | | CN_GAU | |
| | CBS-APNO | JOB_1012_3 | | CN_GAU | |
| Neutral | | | | | |
| T3 = T1; anti(H) | BP86/def-TZVP | job_GEO_277 | job_SP_296 | CN_2 | |
| T3 = T1; syn(H) | BP86/def-TZVP | job_GEO_307 | job_SP_309 | CN_2 | |
| T2 = T4 | BP86/def-TZVP | job_GEO_278 | job_SP_295 | CN_2 | |
| T2 = T5 | BP86/def-TZVP | job_GEO_109 | job_SP_113 | CN_1 | |
| | BP86/def2-TZVPP | job_GEO_543 | job_SP_571 | CN_1 | COSMO-BP-TZVPD-FINE_71 |
| | BP86/def2-TZVPPD | job_GEO_753 | job_SP_805 | CN_1 | |
| | B3LYP/def2-TZVPPD | job_GEO_754 | job_SP_806 | CN_1 | |
| | B3LYP/6-311+G(d,p) | JOB_1013 | | CN_GAU | |
| | G4(MP2) | JOB_1013_1 | | CN_GAU | |
| | W1RO | JOB_1013_2 | | CN_GAU | |

| | | | | | |
|---|---|---|---|---|---|
| | CBS-APNO | JOB_1013_3 | | CN_GAU | |
| T6 | BP86/def-TZVP | job_GEO_110 | job_SP_112 | CN_1 | |
| **8** | | | | | |
| Anion | | | | | |
| syn-syn | BP86/def-TZVP | job_GEO_279 | job_SP_297 | CN_2 | COSMO-BP-TZVPD-FINE_132 |
| anti-syn | BP86/def-TZVP | job_GEO_308 | job_SP_316 | CN_2 | COSMO-BP-TZVPD-FINE_134 |
| | BP86/def2-TZVPP | job_GEO_755 | job_SP_807 | CN_1 | |
| | BP86/def2-TZVPPD | job_GEO_756 | job_SP_808 | CN_1 | |
| | B3LYP/def2-TZVPPD | job_GEO_757 | job_SP_809 | CN_1 | |
| | B3LYP/6-311+G(d,p) | JOB_1014 | | CN_GAU | |
| | G4(MP2) | JOB_1014_1 | | CN_GAU | |
| | CBS-APNO | JOB_1014_3 | | CN_GAU | |
| anti-anti | BP86/def-TZVP | job_GEO_116 | job_SP_117 | CN_1 | |
| | BP86/def2-TZVPP | job_GEO_545 | job_SP_572 | CN_1 | COSMO-BP-TZVPD-FINE_73 |
| Neutral | | | | | |
| T1; syn-syn | BP86/def-TZVP | job_GEO_175 | job_SP_184 | CN_1 | |
| T1; anti-anti | BP86/def-TZVP | job_GEO_114 | job_SP_118 | CN_1 | |
| T1; anti-syn = syn-anti | BP86/def-TZVP | job_GEO_280 | job_SP_298 | CN_2 | |
| T2; anti-anti | BP86/def-TZVP | job_GEO_283 | job_SP_301 | CN_2 | |
| | BP86/def2-TZVPP | job_GEO_546 | job_SP_573 | CN_1 | COSMO-BP-TZVPD-FINE_114 |
| T2; syn-anti | BP86/def-TZVP | job_GEO_317 | job_SP_322 | CN_2 | COSMO-BP-TZVPD-FINE_136 |
| | BP86/def2-TZVPP | job_GEO_810 | job_SP_811 | CN_1 | |
| | BP86/def2-TZVPPD | job_GEO_823 | job_SP_826 | CN_1 | |
| | B3LYP/def2-TZVPPD | job_GEO_824 | job_SP_835 | CN_1 | |
| | B3LYP/6-311+G(d,p) | JOB_1015 | | CN_GAU | |
| | G4(MP2) | JOB_1015_1 | | CN_GAU | |
| | CBS-APNO | JOB_1015_3 | | CN_GAU | |
| T2; syn-syn | BP86/def-TZVP | job_GEO_123 | job_SP_124 | CN_1 | COSMO-BP-TZVPD-FINE_138 |
| T3; cis-anti, anti(H) | BP86/def-TZVP | job_GEO_315 | job_SP_321 | CN_2 | |
| T3; cis-anti, syn(H) | BP86/def-TZVP | job_GEO_152 | job_SP_153 | CN_1 | |
| T3; cis-anti, eclipsed(H) | BP86/def-TZVP | job_GEO_170 | job_SP_171 | CN_1 | |
| T3; trans-anti, syn(H) | BP86/def-TZVP | job_GEO_154 | job_SP_155 | CN_1 | |
| T3; trans-anti, anti(H) | BP86/def-TZVP | job_GEO_162 | job_SP_174 | CN_1 | |
| T3; cis-syn, eclipsed(H) | BP86/def-TZVP | job_GEO_157 | job_SP_167 | CN_1 | |
| T3; trans-syn, syn(H) | BP86/def-TZVP | job_GEO_159 | job_SP_168 | CN_1 | |
| T3; trans-syn, anti(H) | BP86/def-TZVP | job_GEO_161 | job_SP_173 | CN_1 | |
| T4; cis-anti | BP86/def-TZVP | job_GEO_122 | job_SP_125 | CN_1 | |
| T4; cis-syn | BP86/def-TZVP | job_GEO_121 | job_SP_126 | CN_1 | |

| | | | | | |
|---|---|---|---|---|---|
| T4; trans-syn | BP86/def-TZVP | job_GEO_127 | job_SP_128 | CN_1 | |
| T4; trans-anti | BP86/def-TZVP | job_GEO_289 | job_SP_300 | CN_2 | |
| T5; trans-anti | BP86/def-TZVP | job_GEO_129 | job_SP_133 | CN_1 | |
| T5; cis-syn | BP86/def-TZVP | job_GEO_130 | job_SP_134 | CN_1 | |
| T5; cis-anti | BP86/def-TZVP | job_GEO_131 | job_SP_135 | CN_1 | |
| T5; trans-syn | BP86/def-TZVP | job_GEO_132 | job_SP_136 | CN_1 | |
| T6; anti-anti | BP86/def-TZVP | job_GEO_137 | job_SP_142 | CN_1 | |
| T6; anti-syn | BP86/def-TZVP | job_GEO_150 | job_SP_151 | CN_1 | |
| T6; syn-anti | BP86/def-TZVP | job_GEO_141 | job_SP_147 | CN_1 | |
| T6; syn-syn | BP86/def-TZVP | job_GEO_140 | job_SP_149 | CN_1 | |
| **9** | | | | | |
| Anion | | | | | |
| symmetrical | BP86/def-TZVP | job_GEO_285 | job_SP_302 | CN_2 | |
| | BP86/def2-TZVPP | job_GEO_549 | job_SP_574 | CN_1 | COSMO-BP-TZVPD-FINE_63 |
| | BP86/def2-TZVPPD | job_GEO_837 | job_SP_841 | CN_1 | |
| | B3LYP/def2-TZVPPD | job_GEO_838 | job_SP_845 | CN_1 | |
| | B3LYP/6-311+G(d,p) | JOB_1016 | | CN_GAU | |
| | G4(MP2) | JOB_1016_1 | | CN_GAU | |
| asymmetrical | BP86/def-TZVP | job_GEO_327 | job_SP_331 | CN_2 | |
| | BP86/def2-TZVPP | job_GEO_550 | job_SP_575 | CN_1 | COSMO-BP-TZVPD-FINE_65 |
| Neutral | | | | | |
| T1; asymmetrical, anti-syn-syn; H facing backward | BP86/def-TZVP | job_GEO_286 | job_SP_303 | CN_2 | COSMO-BP-TZVPD-FINE_306 |
| T1; symmetrical, syn-syn-syn; H facing backward | BP86/def-TZVP | job_GEO_324 | job_SP_328 | CN_2 | COSMO-BP-TZVPD-FINE_308 |
| T1; symmetrical, anti-anti-anti; H facing backward | BP86/def-TZVP | job_GEO_178 | job_SP_182 | CN_1 | |
| | BP86/def2-TZVPP | job_GEO_547 | job_SP_576 | CN_1 | |
| | BP86/def2-TZVPPD | job_GEO_869 | job_SP_871 | CN_1 | |
| | B3LYP/def2-TZVPPD | job_GEO_840 | job_SP_846 | CN_1 | |
| | B3LYP/6-311+G(d,p) | JOB_1017 | | CN_GAU | |
| | G4(MP2) | JOB_1017_1 | | CN_GAU | |
| T1; asymmetrical, anti-anti-syn; H facing backward | BP86/def-TZVP | job_GEO_177 | job_SP_181 | CN_1 | COSMO-BP-TZVPD-FINE_310 |
| T3; trans-syn-syn, eclipsed(H) | BP86/def-TZVP | job_GEO_287 | job_SP_306 | CN_2 | |
| T3; trans-syn-syn, anti(H) | BP86/def-TZVP | job_GEO_325 | job_SP_329 | CN_2 | |
| T3; trans-syn-syn, syn(H) | BP86/def-TZVP | job_GEO_209 | job_SP_210 | CN_1 | |
| T3; trans-syn-anti, syn(H) | BP86/def-TZVP | job_GEO_211 | job_SP_233 | CN_1 | |
| T3; trans-syn-anti, anti(H) | BP86/def-TZVP | job_GEO_212 | job_SP_223 | CN_1 | |
| T3; trans-syn-anti, eclipsed(H) | BP86/def-TZVP | job_GEO_213 | job_SP_225 | CN_1 | |
| T3; trans-anti-anti, syn(H) | BP86/def-TZVP | job_GEO_220 | job_SP_226 | CN_1 | |
| T3; trans-anti-anti, anti(H) | BP86/def-TZVP | job_GEO_221 | job_SP_234 | CN_1 | |

| | | | | | |
|---|---|---|---|---|---|
| T3; trans-anti-anti, syn(H) (symmetrical) | BP86/def-TZVP | job_GEO_216 | job_SP_228 | CN_1 | |
| T3; trans-syn-anti, syn(H) | BP86/def-TZVP | job_GEO_217 | job_SP_229 | CN_1 | |
| T3; trans-syn-anti, anti(H) | BP86/def-TZVP | job_GEO_218 | job_SP_235 | CN_1 | |
| T3; trans-syn-anti, eclipsed(H) | BP86/def-TZVP | job_GEO_231 | job_SP_232 | CN_1 | |
| T4; trans-anti-syn | BP86/def-TZVP | job_GEO_288 | job_SP_304 | CN_2 | COSMO-BP-TZVPD-FINE_140 |
| T4; trans-syn-anti | BP86/def-TZVP | job_GEO_179 | job_SP_187 | CN_1 | |
| T4; trans-syn-anti | BP86/def2-TZVPP | job_GEO_548 | job_SP_577 | CN_1 | COSMO-BP-TZVPD-FINE_69 |
| T4; trans-syn-syn | BP86/def-TZVP | job_GEO_185 | job_SP_189 | CN_1 | COSMO-BP-TZVPD-FINE_142 |
| T4; trans-anti-anti (symmetrical) | BP86/def-TZVP | job_GEO_183 | job_SP_186 | CN_1 | COSMO-BP-TZVPD-FINE_144 |
| T5; trans-anti-syn | BP86/def-TZVP | job_GEO_193 | job_SP_198 | CN_1 | |
| T5; trans-syn-anti | BP86/def-TZVP | job_GEO_190 | job_SP_199 | CN_1 | |
| T5; trans-syn-syn | BP86/def-TZVP | job_GEO_192 | job_SP_200 | CN_1 | |
| T5; trans-anti-anti (symmetrical) | BP86/def-TZVP | job_GEO_191 | job_SP_207 | CN_1 | |
| T6; anti-anti-syn | BP86/def-TZVP | job_GEO_197 | job_SP_202 | CN_1 | |
| T6; anti-syn-anti | BP86/def-TZVP | job_GEO_194 | job_SP_203 | CN_1 | |
| T6; anti-syn-syn | BP86/def-TZVP | job_GEO_196 | job_SP_206 | CN_1 | |
| T6; anti-anti-anti (symmetrical) | BP86/def-TZVP | job_GEO_195 | job_SP_208 | CN_1 | |
| | | | | | |
| **10** | | | | | |
| Anion | | | | | |
| Pyramidal | BP86/def-TZVP | job_GEO_365 | job_SP_366 | CN_2 | |
| | BP86/def2-TZVPP | job_GEO_551 | job_SP_578 | CN_1 | COSMO-BP-TZVPD-FINE_75 |
| | BP86/def2-TZVPPD | job_GEO_847 | job_SP_872 | CN_1 | |
| | B3LYP/def2-TZVPPD | job_GEO_848 | job_SP_873 | CN_1 | |
| | B3LYP/6-311+G(d,p) | JOB_1018 | | CN_GAU | |
| | G4(MP2) | JOB_1018_1 | | CN_GAU | |
| | W1RO | JOB_1018_2 | | CN_GAU | |
| | CBS-APNO | JOB_1018_3 | | CN_GAU | |
| Neutral | | | | | |
| T2 | BP86/def-TZVP | job_GEO_352 | job_SP_355 | CN_2 | |
| | BP86/def2-TZVPP | job_GEO_552 | job_SP_579 | CN_1 | COSMO-BP-TZVPD-FINE_77 |
| | BP86/def2-TZVPPD | job_GEO_849 | job_SP_874 | CN_1 | |
| | B3LYP/def2-TZVPPD | job_GEO_850 | job_SP_875 | CN_1 | |
| | B3LYP/6-311+G(d,p) | JOB_1019 | | CN_GAU | |
| | G4(MP2) | JOB_1019_1 | | CN_GAU | |
| | W1RO | JOB_1019_2 | | CN_GAU | |
| | CBS-APNO | JOB_1019_3 | | CN_GAU | |
| | | | | | |
| **11** | | | | | |

| | | | | | |
|---|---|---|---|---|---|
| Anion | | | | | |
| | BP86/def-TZVP | job_GEO_356 | jos_SP_390 | CN_2 | |
| | BP86/def2-TZVPP | job_GEO_553 | job_SP_598 | CN_1 | COSMO-BP-TZVPD-FINE_79 |
| | BP86/def2-TZVPPD | job_GEO_851 | job_SP_878 | CN_1 | |
| | B3LYP/def2-TZVPPD | job_GEO_852 | job_SP_879 | CN_1 | |
| | B3LYP/6-311+G(d,p) | JOB_1020 | | CN_GAU | |
| | G4(MP2) | JOB_1020_1 | | CN_GAU | |
| | W1RO | JOB_1020_2 | | CN_GAU | |
| | CBS-APNO | JOB_1020_3 | | CN_GAU | |
| T2; protonated on CN-1 | BP86/def-TZVP | job_GEO_360 | job_SP_392 | CN_2 | |
| T2; protonated on CN-2 | BP86/def-TZVP | job_GEO_238 | job_SP_242 | CN_1 | |
| T2; protonated on CN-3 | BP86/def-TZVP | job_GEO_239 | job_SP_243 | CN_1 | |
| | BP86/def2-TZVPP | job_GEO_554 | job_SP_599 | CN_1 | COSMO-BP-TZVPD-FINE_81 |
| | BP86/def2-TZVPPD | job_GEO_853 | job_SP_880 | CN_1 | |
| | B3LYP/def2-TZVPPD | job_GEO_854 | job_SP_881 | CN_1 | |
| | B3LYP/6-311+G(d,p) | JOB_1021 | | CN_GAU | |
| | G4(MP2) | JOB_1021_1 | | CN_GAU | |
| | W1RO | JOB_1021_2 | | CN_GAU | |
| | CBS-APNO | JOB_1021_3 | | CN_GAU | |
| T4 | BP86/def-TZVP | job_GEO_244 | job_SP_245 | CN_1 | |
| T5 | BP86/def-TZVP | job_GEO_389 | job_SP_391 | CN_1 | |
| T6 | BP86/def-TZVP | job_GEO_237 | job_SP_241 | CN_1 | |
| **12** | | | | | |
| Anion | | | | | |
| anti-syn; asymmetric | BP86/def-TZVP | job_GEO_454 | job_SP_457 | CN_2 | |
| | BP86/def2-TZVPP | job_GEO_555 | job_SP_600 | CN_1 | COSMO-BP-TZVPD-FINE_83 |
| | BP86/def2-TZVPPD | job_GEO_855 | job_SP_882 | CN_1 | |
| | B3LYP/def2-TZVPPD | job_GEO_856 | job_SP_883 | CN_1 | |
| | B3LYP/6-311+G(d,p) | JOB_1022 | | CN_GAU | |
| | G4(MP2) | JOB_1022_1 | | CN_GAU | |
| | CBS-APNO | JOB_1022_3 | | CN_GAU | |
| syn-syn; symmetric | BP86/def-TZVP | job_GEO_455 | job_SP_458 | CN_2 | COSMO-BP-TZVPD-FINE_147 |
| anti-anti; symmetric | BP86/def-TZVP | job_GEO_456 | job_SP_459 | CN_2 | COSMO-BP-TZVPD-FINE_149 |
| Neutral | | | | | |
| T2; protonated on CN-1; anti-syn | BP86/def-TZVP | job_GEO_114 | job_SP_115 | CN_3 | |
| | BP86/def2-TZVPP | job_GEO_556 | job_SP_601 | CN_1 | COSMO-BP-TZVPD-FINE_85 |
| | BP86/def2-TZVPPD | job_GEO_857 | job_SP_884 | CN_1 | |
| | B3LYP/def2-TZVPPD | job_GEO_858 | job_SP_885 | CN_1 | |

| | | | | | |
|---|---|---|---|---|---|
| | B3LYP/6-311+G(d,p) | JOB_1023 | | CN_GAU | |
| | G4(MP2) | JOB_1023 _1 | | CN_GAU | |
| | CBS-APNO | JOB_1023_3 | | CN_GAU | |
| T2; protonated on CN-1; syn-syn | BP86/def-TZVP | job_GEO_253 | job_SP_304 | CN_1 | COSMO-BP-TZVPD-FINE_151 |
| T2; protonated on CN-1; rather anti-syn | BP86/def-TZVP | job_GEO_252 | job_SP_305 | CN_1 | COSMO-BP-TZVPD-FINE_167 |
| T2; protonated on CN-2; syn-syn | BP86/def-TZVP | job_GEO_255 | job_SP_306 | CN_1 | |
| T2; protonated on CN-2; anti-syn | BP86/def-TZVP | job_GEO_250 | job_SP_307 | CN_1 | |
| T2; protonated on CN-2; syn-anti | BP86/def-TZVP | job_GEO_251 | job_SP_308 | CN_1 | |
| T2; protonated on CN-2; anti-anti | BP86/def-TZVP | job_GEO_254 | job_SP_309 | CN_1 | COSMO-BP-TZVPD-FINE_169 |
| T4; cis-anti | BP86/def-TZVP | job_GEO_113 | job_SP_116 | CN_3 | |
| T4; cis-syn | BP86/def-TZVP | job_GEO_258 | job_SP_267 | CN_1 | |
| T4; trans-anti | BP86/def-TZVP | job_GEO_256 | job_SP_295 | CN_1 | |
| T4; trans-syn | BP86/def-TZVP | job_GEO_257 | job_SP_296 | CN_1 | |
| T5; cis-anti | BP86/def-TZVP | job_GEO_261 | job_SP_297 | CN_1 | |
| T5; cis-anti | BP86/def-TZVP | job_GEO_262 | job_SP_283 | CN_1 | |
| T5; cis-syn | BP86/def-TZVP | job_GEO_286 | job_SP_298 | CN_1 | |
| T5; trans-syn | BP86/def-TZVP | job_GEO_259 | job_SP_299 | CN_1 | |
| T5; trans-anti | BP86/def-TZVP | job_GEO_260 | job_SP_300 | CN_1 | |
| T6; anti-syn | BP86/def-TZVP | job_GEO_264 | job_SP_301 | CN_1 | |
| T6; syn-syn | BP86/def-TZVP | job_GEO_266 | job_SP_302 | CN_1 | |
| T6; anti-anti | BP86/def-TZVP | job_GEO_268 | job_SP_281 | CN_1 | |
| T6; syn-anti | BP86/def-TZVP | job_GEO_265 | job_SP_281 | CN_1 | |
| **13** | | | | | |
| Anion | | | | | |
| anti-syn-anti | BP86/def-TZVP | job_GEO_129 | job_SP_136 | CN_3 | COSMO-BP-TZVPD-FINE_155 |
| anti-syn-syn | BP86/def-TZVP | job_GEO_131 | job_SP_138 | CN_3 | COSMO-BP-TZVPD-FINE_157 |
| syn-syn-syn | BP86/def-TZVP | job_GEO_248 | job_SP_249 | CN_1 | COSMO-BP-TZVPD-FINE_153 |
| anti-anti-anti | BP86/def-TZVP | job_GEO_137 | job_SP_139 | CN_3 | |
| | BP86/def2-TZVPP | job_GEO_557 | job_SP_604 | CN_1 | COSMO-BP-TZVPD-FINE_87 |
| | BP86/def2-TZVPPD | job_GEO_859 | job_SP_886 | CN_1 | |
| | B3LYP/def2-TZVPPD | job_GEO_860 | job_SP_887 | CN_1 | |
| | B3LYP/6-311+G(d,p) | JOB_1024 | | CN_GAU | |
| | G4(MP2) | JOB_1024 _1 | | CN_GAU | |
| Neutral | | | | | |
| T2, anti-anti-syn | BP86/def-TZVP | job_GEO_127 | job_SP_134 | CN_3 | COSMO-BP-TZVPD-FINE_159 |
| T2, anti-anti-anti | BP86/def-TZVP | job_GEO_100 | job_SP_106 | CN_3 | COSMO-BP-TZVPD-FINE_161 |
| | BP86/def2-TZVPP | job_GEO_558 | job_SP_603 | CN_1 | COSMO-BP-TZVPD-FINE_89 |
| | BP86/def2-TZVPPD | job_GEO_861 | job_SP_888 | CN_1 | |

| | | | | | |
|---|---|---|---|---|---|
| | B3LYP/def2-TZVPPD | job_GEO_862 | job_SP_889 | CN_1 | |
| | B3LYP/6-311+G(d,p) | JOB_1025 | | | |
| | G4(MP2) | JOB_1025 _1 | | | |
| T2, anti-syn-syn | BP86/def-TZVP | job_GEO_313 | job_SP_314 | CN_1 | COSMO-BP-TZVPD-FINE_163 |
| T2, syn-syn-syn | BP86/def-TZVP | job_GEO_312 | job_SP_315 | CN_1 | COSMO-BP-TZVPD-FINE_165 |
| T4, trans-syn-anti | BP86/def-TZVP | job_GEO_128 | job_SP_135 | CN_3 | |
| T4; cis-syn-anti | BP86/def-TZVP | job_GEO_317 | job_SP_366 | CN_1 | |
| T4; trans-syn-syn | BP86/def-TZVP | job_GEO_318 | job_SP_367 | CN_1 | |
| T4; cis-syn-syn (symmetrical) | BP86/def-TZVP | job_GEO_319 | job_SP_373 | CN_1 | |
| T4; trans-anti-anti (symmetrical) | BP86/def-TZVP | job_GEO_320 | job_SP_374 | CN_1 | |
| T4; cis-anti-anti | BP86/def-TZVP | job_GEO_321 | job_SP_375 | CN_1 | |
| T4; trans-anti-syn | BP86/def-TZVP | job_GEO_322 | job_SP_371 | CN_1 | |
| T4; cis-anti-syn | BP86/def-TZVP | job_GEO_323 | job_SP_372 | CN_1 | |
| T5; trans-(anti)-anti | BP86/def-TZVP | job_GEO_340 | job_SP_377 | CN_1 | |
| T5; cis-syn-anti | BP86/def-TZVP | job_GEO_333 | job_SP_378 | CN_1 | |
| T5; trans-(anti)-syn | BP86/def-TZVP | job_GEO_334 | job_SP_383 | CN_1 | |
| T5; cis-syn-(syn) | BP86/def-TZVP | job_GEO_343 | job_SP_380 | CN_1 | |
| T5; trans-anti-anti | BP86/def-TZVP | job_GEO_336 | job_SP_381 | CN_1 | |
| T5; cis-anti-anti | BP86/def-TZVP | job_GEO_337 | job_SP_382 | CN_1 | |
| T5; trans-anti-syn | BP86/def-TZVP | job_GEO_338 | job_SP_392 | CN_1 | |
| T5; cis-anti-(anti) | BP86/def-TZVP | job_GEO_347 | job_SP_385 | CN_1 | |
| T6; trans-syn-anti | BP86/def-TZVP | job_GEO_348 | job_SP_402 | CN_1 | |
| T6; cis-(anti)-anti | BP86/def-TZVP | job_GEO_341 | job_SP_394 | CN_1 | |
| T6; trans-syn-(syn) | BP86/def-TZVP | job_GEO_342 | job_SP_403 | CN_1 | |
| T6; cis-syn-syn (symmetrical) | BP86/def-TZVP | job_GEO_335 | job_SP_401 | CN_1 | |
| T6; trans-anti-anti (symmetrical) | BP86/def-TZVP | job_GEO_344 | job_SP_404 | CN_1 | |
| T6; cis-anti-anti | BP86/def-TZVP | job_GEO_345 | job_SP_398 | CN_1 | |
| T6; trans-anti-(anti) | BP86/def-TZVP | job_GEO_346 | job_SP_405 | CN_1 | |
| T6; cis-ani-syn | BP86/def-TZVP | job_GEO_339 | job_SP_400 | CN_1 | |
| **14** | | | | | |
| Anion | | | | | |
| conformer I | BP86/def-TZVP | job_GEO_86 | job_SP_88 | CN_3 | COSMO-BP-TZVPD-FINE_175 |
| conformer II | BP86/def-TZVP | job_GEO_406 | job_SP_421 | CN_1 | |
| | BP86/def2-TZVPP | job_GEO_559 | job_SP_595 | CN_1 | COSMO-BP-TZVPD-FINE_91 |
| conformer III; most stable from CosmoConf | BP86/def-TZVP | job_GEO_675 | job_SP_676 | CN_1 | COSMO-BP-TZVPD-FINE_171 |
| | BP86/def2-TZVPP | job_GEO_863 | job_SP_890 | CN_1 | |
| | BP86/def2-TZVPPD | job_GEO_864 | job_SP_891 | CN_1 | |
| | B3LYP/6-311+G(d,p) | JOB_1026 | | CN_GAU | |

| | | | | | |
|---|---|---|---|---|---|
| | G4(MP2) | JOB_1026 _1 | | CN_GAU | |
| conformer IV | BP86/def-TZVP | job_GEO_677 | job_SP_680 | CN_1 | COSMO-BP-TZVPD-FINE_173 |
| Neutral | | | | | |
| T4; conformer I | BP86/def-TZVP | job_GEO_109 | job_SP_117 | CN_3 | |
| T4; conformer I | BP86/def-TZVP | job_GEO_437 | job_SP_449 | CN_1 | |
| T4; conformer I | BP86/def-TZVP | job_GEO_440 | job_SP_450 | CN_1 | |
| T4; conformer I | BP86/def-TZVP | job_GEO_443 | job_SP_451 | CN_1 | |
| T4; conformer I | BP86/def-TZVP | job_GEO_446 | job_SP_452 | CN_1 | |
| T4; conformer II | BP86/def-TZVP | job_GEO_410 | job_SP_424 | CN_1 | |
| T4; conformer II | BP86/def-TZVP | job_GEO_422 | job_SP_433 | CN_1 | |
| T4; conformer II | BP86/def-TZVP | job_GEO_416 | job_SP_429 | CN_1 | |
| T4; conformer II | BP86/def-TZVP | job_GEO_419 | job_SP_434 | CN_1 | |
| T4; conformer III | BP86/def-TZVP | CN_684 (14_AN_1_T4_1) | job_SP_685 | CN_1 | |
| T4; conformer III | BP86/def-TZVP | CN_684 (14_AN_1_T4_2) | job_SP_686 | CN_1 | |
| T4; conformer III | BP86/def-TZVP | CN_684 (14_AN_1_T4_3) | job_SP_687 | CN_1 | |
| T4; conformer III | BP86/def-TZVP | CN_684 (14_AN_1_T4_4) | job_SP_688 | CN_1 | |
| T4; conformer IV | BP86/def-TZVP | CN_697 (14_AN_2_T4_1) | job_SP_698 | CN_1 | |
| T4; conformer IV | BP86/def-TZVP | CN_697 (14_AN_2_T4_2) | job_SP_699 | CN_1 | |
| T4; conformer IV | BP86/def-TZVP | CN_697 (14_AN_2_T4_3) | job_SP_700 | CN_1 | |
| T4; conformer IV | BP86/def-TZVP | CN_697 (14_AN_2_T4_4) | job_SP_701 | CN_1 | |
| T5; conformer I | BP86/def-TZVP | job_GEO_448 | job_SP_453 | CN_1 | |
| T5; conformer I | BP86/def-TZVP | job_GEO_465 | job_SP_466 | CN_1 | |
| T5; conformer I | BP86/def-TZVP | job_GEO_442 | job_SP_461 | CN_1 | |
| T5; conformer I | BP86/def-TZVP | job_GEO_447 | job_SP_456 | CN_1 | |
| T5; conformer II | BP86/def-TZVP | job_GEO_409 | job_SP_423 | CN_1 | |
| T5; conformer II | BP86/def-TZVP | job_GEO_414 | job_SP_427 | CN_1 | |
| T5; conformer II | BP86/def-TZVP | job_GEO_415 | job_SP_430 | CN_1 | |
| T5; conformer II | BP86/def-TZVP | job_GEO_418 | job_SP_432 | CN_1 | |
| T5; conformer III | BP86/def-TZVP | CN_684 (14_AN_1_T5_1) | job_SP_689 | CN_1 | |
| T5; conformer III | BP86/def-TZVP | CN_684 (14_AN_1_T5_2) | job_SP_690 | CN_1 | |
| T5; conformer III | BP86/def-TZVP | CN_684 (14_AN_1_T5_3) | job_SP_691 | CN_1 | |
| T5; conformer III | BP86/def-TZVP | CN_684 (14_AN_1_T5_4) | job_SP_692 | CN_1 | |
| T5; conformer IV | BP86/def-TZVP | CN_697 (14_AN_2_T5_1) | job_SP_702 | CN_1 | |
| T5; conformer IV | BP86/def-TZVP | CN_697 (14_AN_2_T5_2) | job_SP_703 | CN_1 | |
| T5; conformer IV | BP86/def-TZVP | CN_697 (14_AN_2_T5_3) | job_SP_704 | CN_1 | |
| T5; conformer IV | BP86/def-TZVP | CN_697 (14_AN_2_T5_4) | job_SP_705 | CN_1 | |
| T6; conformer I | BP86/def-TZVP | job_GEO_438 | job_SP_457 | CN_1 | |
| T6; conformer I | BP86/def-TZVP | job_GEO_441 | job_SP_462 | CN_1 | |
| T6; conformer I | BP86/def-TZVP | job_GEO_444 | job_SP_463 | CN_1 | COSMO-BP-TZVPD-FINE_178 |

| | | | | | |
|---|---|---|---|---|---|
| T6; conformer I | BP86/def-TZVP | job_GEO_445 | job_SP_464 | CN_1 | |
| T6; conformer II | BP86/def-TZVP | job_GEO_411 | job_SP_425 | CN_1 | |
| T6; conformer II | BP86/def-TZVP | job_GEO_412 | job_SP_426 | CN_1 | |
| T6; conformer II | BP86/def2-TZVPP | job_GEO_560 | job_SP_602 | CN_1 | COSMO-BP-TZVPD-FINE_93 |
| T6; conformer II | BP86/def-TZVP | job_GEO_417 | job_SP_431 | CN_1 | |
| T6; conformer II | BP86/def-TZVP | job_GEO_420 | job_SP_435 | CN_1 | |
| T6; conformer III | BP86/def-TZVP | CN_684 (14_AN_1_T6_1) | job_SP_693 | CN_1 | COSMO-BP-TZVPD-FINE_180 |
| T6; conformer III | BP86/def-TZVP | CN_684 (14_AN_1_T6_2) | job_SP_694 | CN_1 | |
| T6; conformer III | BP86/def-TZVP | CN_684 (14_AN_1_T6_3) | job_SP_695 | CN_1 | |
| T6; conformer III | BP86/def2-TZVPP | job_GEO_866 | job_SP_892 | CN_1 | |
| T6; conformer III | BP86/def2-TZVPPD | job_GEO_867 | job_SP_893 | CN_1 | |
| T6; conformer III | B3LYP/6-311+G(d,p) | JOB_1027 | | CN_GAU | |
| T6; conformer III | G4(MP2) | JOB_1027 _1 | | CN_GAU | |
| T6; conformer III | BP86/def-TZVP | CN_684 (14_AN_1_T6_4) | job_SP_696 | CN_1 | |
| T6; conformer IV | BP86/def-TZVP | CN_697 (14_AN_2_T6_1) | job_SP_706 | CN_1 | |
| T6; conformer IV | BP86/def-TZVP | CN_697 (14_AN_2_T6_2) | job_SP_707 | CN_1 | |
| T6; conformer IV | BP86/def-TZVP | CN_697 (14_AN_2_T6_3) | job_SP_708 | CN_1 | COSMO-BP-TZVPD-FINE_182 |
| T6; conformer IV | BP86/def-TZVP | CN_697 (14_AN_2_T6_4) | job_SP_709 | CN_1 | |
| | | | | | |
| **15** | | | | | |
| Anion | | | | | |
| | BP86/def-TZVP | job_GEO_539 | job_SP_545 | CN_3 | |
| | B3LYP/def2-TZVPPD | job_GEO_561 | job_SP_605 | CN_1 | COSMO-BP-TZVPD-FINE_95 |
| | B3LYP/6-311+G(d,p) | JOB_1029 | | CN_GAU | |
| Neutral | | | | | |
| C-N-H | BP86/def-TZVP | job_GEO_540 | job_SP_546 | CN_3 | |
| H-C-N | BP86/def-TZVP | job_GEO_541 | job_SP_547 | CN_3 | |
| | B3LYP/def2-TZVPPD | job_GEO_562 | job_SP_606 | CN_1 | COSMO-BP-TZVPD-FINE_97 |
| | B3LYP/6-311+G(d,p) | JOB_1030 | | CN_GAU | |
| | | | | | |
| **16** | | | | | |
| Anion | | | | | |
| | BP86/def-TZVP | job_GEO_542 | job_SP_548 | CN_3 | |
| | B3LYP/def2-TZVPPD | job_GEO_563 | job_SP_607 | CN_1 | COSMO-BP-TZVPD-FINE_99 |
| | B3LYP/6-311+G(d,p) | JOB_1031 | | CN_GAU | |
| Neutal | | | | | |
| T1; C(CN)2=CH(CN) | BP86/def-TZVP | job_GEO_552 | job_SP_553 | CN_3 | |
| | B3LYP/def2-TZVPPD | job_GEO_564 | job_SP_608 | CN_1 | COSMO-BP-TZVPD-FINE_101 |
| | B3LYP/6-311+G(d,p) | JOB_1032 | | CN_GAU | |

| | | | | | |
|---|---|---|---|---|---|
| T4; C(CN-H)2=C(CN) | BP86/def-TZVP | job_GEO_544 | job_SP_550 | CN_3 | |
| T6; C(CN)2=C(CN-H) | BP86/def-TZVP | job_GEO_477 | job_SP_483 | CN_1 | |
| T5; C(CN-H)2=C(CN) | BP86/def-TZVP | job_GEO_478 | job_SP_484 | CN_1 | |
| | | | | | |
| **17** | | | | | |
| Dianion | | | | | |
| [B12(CN)12]2- | BP86/def-TZVP | job_GEO_364 | job_SP_369 | CN_3 | |
| | BP86/def-TZVP | job_GEO_486 | job_SP_487 | CN_1 | |
| | B3LYP/def2-TZVPPD | job_GEO_565 | job_SP_594 | CN_1 | COSMO-BP-TZVPD-FINE_103 |
| | B3LYP/6-311+G(d,p) | JOB_1033 | | CN_GAU | |
| Anion | | | | | |
| [B12(CN)12]-, protonated on CN | BP86/def-TZVP | job_GEO_365 | job_SP_370 | CN_3 | |
| | B3LYP/def2-TZVPPD | job_GEO_566 | job_SP_597 | CN_1 | COSMO-BP-TZVPD-FINE_105 |
| | B3LYP/6-311+G(d,p) | JOB_1034 | | CN_GAU | |
| | | | | | |
| **18** | | | | | |
| Neutral | | | | | |
| B12(CN)12H2, H-s dide by side | BP86/def-TZVP | job_GEO_373 | job_SP_379 | CN_3 | |
| B12(CN)12H2, 1 B between two H-s | BP86/def-TZVP | job_GEO_376 | job_SP_380 | CN_3 | |
| | BP86/def-TZVP | job_GEO_488 | job_SP_537 | CN_1 | |
| B12(CN)12H2, 2 B-s between two H-s | BP86/def-TZVP | job_GEO_372 | job_SP_381 | CN_3 | |
| | B3LYP/def2-TZVPPD | job_GEO_567 | job_SP_593 | CN_1 | COSMO-BP-TZVPD-FINE_107 |
| | B3LYP/6-311+G(d,p) | JOB_1035 | | CN_GAU | |
| | | | | | |
| **19** | | | | | |
| Anion | | | | | |
| [CB11(CN)12]- | BP86/def-TZVP | job_GEO_391 | job_SP_396 | CN_3 | |
| | B3LYP/def2-TZVPPD | job_GEO_568 | job_SP_592 | CN_1 | COSMO-BP-TZVPD-FINE_109 |
| | B3LYP/6-311+G(d,p) | JOB_1036 | | CN_GAU | |
| Neutral | | | | | |
| CB11(CN)12-H, H on CN that is on C | BP86/def-TZVP | job_GEO_392 | job_SP_397 | CN_3 | |
| CB11(CN)12-H, H on CN that is on 1st row B | BP86/def-TZVP | job_GEO_393 | job_SP_398 | CN_3 | |
| CB11(CN)12-H, H on CN that is on 2nd row B | BP86/def-TZVP | job_GEO_394 | job_SP_399 | CN_3 | |
| CB11(CN)12-H, H on CN that is on opposite C | BP86/def-TZVP | job_GEO_395 | job_SP_400 | CN_3 | |
| | B3LYP/def2-TZVPPD | job_GEO_569 | job_SP_596 | CN_1 | COSMO-BP-TZVPD-FINE_111 |
| | B3LYP/6-311+G(d,p) | JOB_1037 | | CN_GAU | |

**Table S11.** DataDOI data repository filenames of auxiliary acids in the COSMO-RS folder. See http://dx.doi.org/10.23673/re-436.

| Compd name | CAS | | FILENAME | | |
|---|---|---|---|---|---|
| | | | ANION | | NEUTRAL |
| $NH_2$-TCNP | 98555-43-2 | | COSMO-BP-TZVPD-FINE_184 | T4 | COSMO-BP-TZVPD-FINE_186 |
| | | | | T5 | COSMO-BP-TZVPD-FINE_188 |
| | | | | T3 | COSMO-BP-TZVPD-FINE_190 |
| Me-TCNP | 24943-63-3 | | COSMO-BP-TZVPD-FINE_192 | T4 | COSMO-BP-TZVPD-FINE_194 |
| | | | | T5 | COSMO-BP-TZVPD-FINE_196 |
| 3,4-$(MeO)_2$-$C_6H_3$-TCNP | 1261286-75-2 | Conf 1 | COSMO-BP-TZVPD-FINE_198 | T4 | COSMO-BP-TZVPD-FINE_289 |
| | | | | T5 | COSMO-BP-TZVPD-FINE_202 |
| | | Conf 2 | | T4 | COSMO-BP-TZVPD-FINE_204 |
| | | | | T5 | COSMO-BP-TZVPD-FINE_304 |
| 4-MeO-$C_6H_4$-TCNP | 1261286-76-3 | | COSMO-BP-TZVPD-FINE_208 | T4 | COSMO-BP-TZVPD-FINE_210 |
| | | | | T5 | COSMO-BP-TZVPD-FINE_212 |
| Ph-TCNP | 96463-47-7 | | COSMO-BP-TZVPD-FINE_214 | T4 | COSMO-BP-TZVPD-FINE_216 |
| | | | | T5 | COSMO-BP-TZVPD-FINE_219 |
| 3-$CF_3$-$C_6H_4$-TCNP | 2226486-85-5 | Conf 1 | COSMO-BP-TZVPD-FINE_223 | T4 | COSMO-BP-TZVPD-FINE_225 |
| | | | | T5 | COSMO-BP-TZVPD-FINE_227 |
| | | Conf 2 | | T4 | COSMO-BP-TZVPD-FINE_229 |
| | | | | T5 | COSMO-BP-TZVPD-FINE_233 |
| H-TCNP | 32019-26-4 | | COSMO-BP-TZVPD-FINE_235 | T3 | COSMO-BP-TZVPD-FINE_237 |
| | | | | T4 | COSMO-BP-TZVPD-FINE_239 |
| Br-TCNP | 100125-09-5 | | COSMO-BP-TZVPD-FINE_243 | T4 | COSMO-BP-TZVPD-FINE_245 |
| | | | | T5 | COSMO-BP-TZVPD-FINE_247 |
| 3,5-$(CF_3)_2$-$C_6H_3$-TCNP | 1261286-79-6 | | COSMO-BP-TZVPD-FINE_249 | T4 | COSMO-BP-TZVPD-FINE_251 |
| | | | | T5 | COSMO-BP-TZVPD-FINE_253 |
| Cl-TCNP | 1261286-80-9 | | COSMO-BP-TZVPD-FINE_256 | T4 | COSMO-BP-TZVPD-FINE_258 |
| | | | | T5 | COSMO-BP-TZVPD-FINE_260 |
| CN-$CH_2$-TCNP | 2638-14-4 | | COSMO-BP-TZVPD-FINE_274 | T4 | COSMO-BP-TZVPD-FINE_276 |
| | | | | T5 | COSMO-BP-TZVPD-FINE_278 |
| $CF_3$-TCNP | 1261286-81-0 | | COSMO-BP-TZVPD-FINE_221 | T4 | COSMO-BP-TZVPD-FINE_264 |
| | | | COSMO-BP-TZVPD-FINE_262 | T5 | COSMO-BP-TZVPD-FINE_266 |
